# Fast Sequences of Non-spatial State Representations in Humans

**DOI:** 10.1016/j.neuron.2016.05.028

**Published:** 2016-07-06

**Authors:** Zeb Kurth-Nelson, Marcos Economides, Raymond J. Dolan, Peter Dayan

**Affiliations:** 1 Max Planck-UCL Centre for Computational Psychiatry and Ageing Research, University College London, London WC1B 5EH, UK; 2 Wellcome Trust Centre for Neuroimaging, University College London, London WC1N 3BG, UK; 3 Gatsby Computational Neuroscience Unit, University College London, London W1T 4JG, UK

## Abstract

Fast internally generated sequences of neural representations are suggested to support learning and online planning. However, these sequences have only been studied in the context of spatial tasks and never in humans. Here, we recorded magnetoencephalography (MEG) while human subjects performed a novel non-spatial reasoning task. The task required selecting paths through a set of six visual objects. We trained pattern classifiers on the MEG activity elicited by direct presentation of the visual objects alone and tested these classifiers on activity recorded during periods when no object was presented. During these object-free periods, the brain spontaneously visited representations of approximately four objects in fast sequences lasting on the order of 120 ms. These sequences followed backward trajectories along the permissible paths in the task. Thus, spontaneous fast sequential representation of states can be measured non-invasively in humans, and these sequences may be a fundamental feature of neural computation across tasks.

## Highlights

• Task requires navigating between objects whose neural representations can be decoded
• After task has been learned, spontaneous MEG activity encodes reverse paths
• These spontaneous sequences are up to four states long
• Sequences might be cognate with rodent hippocampal “replay”

## Teaser

The rat hippocampus spontaneously imagines past and future locations in rapid sequences (sometimes called “replay”), which link multiple locations into coherent neural representations. Kurth-Nelson et al. find similar sequences in humans, in a non-spatial task, suggesting they are a ubiquitous mechanism.

## Introduction

Most areas of the brain are engaged in encoding and representing current sensory inputs, contexts, and motor outputs. However, neural activity can also be decoupled from current input to encode representations of past or possible future states. Such decoupling is argued to underpin memory, imagination, and planning (Buckner and Carroll, 2007, Buzsáki and Moser, 2013, Carr et al., 2011, Jadhav et al., 2012, van der Meer et al., 2012, Pezzulo et al., 2014, Pfeiffer and Foster, 2013, Wikenheiser and Redish, 2015).

A remarkable, but now well-established, finding is that the decoupled activity of populations of neurons sometimes takes the form of internally generated sequences that encode trajectories through past or possible future states. This phenomenon has been most studied in rodent hippocampus, where place cells that normally encode an organism’s current position in space also spontaneously play out sequences of other positions (Diba and Buzsáki, 2007, Foster and Wilson, 2006, Louie and Wilson, 2001, Skaggs and McNaughton, 1996). Internally generated hippocampal sequences occur in two distinct physiological contexts, embedded within sharp-wave ripple events (e.g., Diba and Buzsáki, 2007) or nested within theta rhythm (e.g., Johnson and Redish, 2007). The relationship between sequences in these two contexts remains unknown (Schmidt and Redish, 2013), and here we discuss observations in both. Spontaneous hippocampal sequences have been observed in sleep and wakefulness and appear in a variety of spatial tasks (Davidson et al., 2009, Gupta et al., 2010, Karlsson and Frank, 2009, Lee and Wilson, 2002). Although less extensive, there is also evidence for fast spontaneous sequences outside of hippocampus (Euston et al., 2007, Hoffman and McNaughton, 2002). The interaction of cortical with hippocampal sequences is not yet understood, although in simultaneous recordings the hippocampus plays out what appears to be the same experience as visual cortex (Ji and Wilson, 2007).

Two, not mutually exclusive, classes of function are suggested for fast spontaneous sequences. First, in the context of learning, they may be part of a mechanism for consolidating or maintaining knowledge, particularly in cortex (Káli and Dayan, 2004, Louie and Wilson, 2001, Mnih et al., 2015, Siapas and Wilson, 1998). Temporal compression of sequences, relative to real experience, might bring distal events within a time frame within which synaptic plasticity mechanisms can operate, particularly those used for credit assignment (Foster and Wilson, 2006, Jensen and Lisman, 2005, Skaggs et al., 1996). Second, sequences may play a role in planning or look-ahead in decision making, either online or offline (Sutton, 1991). Sequences beginning at the animal’s current location sometimes predict the path the animal will run in the immediate future (Pfeiffer and Foster, 2013, Wikenheiser and Redish, 2015). Concurrently, value signals emerge that are suggested to be a downstream consequence of such prospection (Lansink et al., 2009, van der Meer and Redish, 2009, van der Meer et al., 2010).

Despite the importance of fast spontaneous sequences, and their potential generality as a mechanism for learning and decision making (Buzsáki and Moser, 2013), they have so far only been studied in spatial tasks, and overwhelmingly in rodents. Our goal was to investigate spontaneous neural sequences in a non-spatial context in healthy human volunteers. Previously, we used multivariate analysis of magnetoencephalography (MEG) data to decode time-resolved representations of visual objects that were not currently being experienced (Kurth-Nelson et al., 2015). We therefore reasoned that it might be possible to detect spontaneous fast sequences using MEG in a non-spatial task in which states were defined by decodeable visual objects.

## Results

### Task

**Figure 1:**
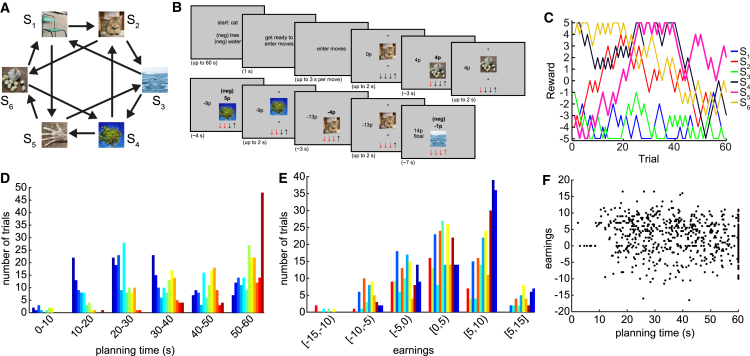
Task Design Participants navigated between six states (S_1_–S_6_), each corresponding to a visual object. (A) The states were linked to one another as shown, although the visual objects assigned to each state number were randomized across participants. (B) On each trial, participants began in a random state and were permitted four moves. They had up to 60 s to plan these four moves. The four moves were then entered rapidly with no feedback. After rapidly entering their chosen sequence of moves, participants were required to play out this sequence. While playing out the sequence, the objects and their associated reward were visible. (C) The reward associated with each state drifted slowly over trials. The total reward earned in each trial was cumulative of the reward collected along the path. When a “neg” state was reached, it caused the sign of the cumulative collected reward to flip (negative to positive and vice versa). (D) Distributions over participants of time used to plan, up to a maximum permitted 60 s. Each color corresponds to a unique participant, sorted by mean planning time. (E) Distributions over participants of money earned per trial, relative to the expected earnings of a random policy on that trial. Each color corresponds to the same participant as in (D). (F) Trials with greater earnings tended to have shorter planning time (p = 0.002 by linear mixed model). However, participants with shorter mean planning time did not have higher mean earnings. Each point is a trial, with all participants shown together.

Participants performed a novel six-state non-spatial navigation task. Each state was defined by a unique visual object and associated with a varying amount of reward, ranging from −5 to +5 pence. From each state, two choices were available (called “up” and “down”), each of which led to a different state (Figures 1A and 1B). Before scanning, participants were trained to criterion on the structure of the task. On each trial during scanning, participants started from a random state and were asked to enter a sequence of four moves with the goal of collecting as much reward as possible. This sequence of moves defined a path around the maze. To discourage simple stimulus-response learning strategies, the task had two additional features. First, reward amounts changed by −1p, 0p, or 1p at random per trial (Figure 1C). Second, at the beginning of each trial, participants were informed that two (randomly selected) states would be “neg,” meaning that reaching either of these states would multiply the trial’s cumulative reward by −1. Importantly, participants never saw a bird’s-eye view of the maze and only experienced one visual object at a time. At debriefing, no participant reported conceiving the relationships between objects in a spatial manner.

We reasoned that neural sequences following the transition structure of the task could only occur if participants in fact learned the transition structure. Although the training and task were designed to encourage such learning, we sought to confirm that it did actually occur. First, in 2–3 days of training before scanning, all participants were required to reach a criterion of 100% accuracy on a set of automated quiz questions that probed knowledge of the transition structure (e.g., “if you start at horn and go up, where will you be?”). Second, in debriefing post scanning, all participants reported a subjective experience of deploying knowledge of transitions for planning. For example, “I didn’t always manage to think four steps ahead, but sometimes I did” and “I tried to make a four-step plan, but sometimes missed the negative on the fourth step.” Third, model comparison on behavioral choice data strongly favored models that planned with the task’s structure over stimulus-response models (Figure S1).

After the starting state was revealed at the beginning of a trial, participants were allowed up to 60 s for planning, with the possibility of entering moves earlier by pressing a button. The distribution of actual planning times is shown in Figure 1D, with a median of 38.9 s (interquartile range [IQR] = 27.3 s). For more details of the timings within each trial, see Experimental Procedures.

After the planning period, participants had to pre-enter their four moves quickly or face a monetary penalty. During this pre-entry, the sole feedback was the appearance of an “up” or “down” arrow, displaying each move that they selected. The mean reaction time to pre-enter the first move in the slowest participant was 760 ± 50 ms and in the fastest participant was 304 ± 21 ms. After pre-entry, participants were required to execute the same sequence of moves they had just pre-entered. During execution, the visual objects associated with each state were displayed, and as they executed each move, the visual object representing the current state faded into the object representing the next state. The mean reaction time to execute the first move in the slowest participant was 998 ± 63 ms and in the fastest participant was 408 ± 18 ms. As each visual object appeared during execution, its current reward value (from −5 to +5 pence) appeared alongside it. The cumulative total trial earnings were displayed continuously during the execution phase and were updated as each visual object appeared.

Task performance, in terms of money earned, was significantly higher than chance for 9/12 participants (Figure 1E). Between trials, there was a significant relationship between planning time and earnings, with more money earned on trials with shorter planning time (Figure 1F; p = 0.002 by mixed effects linear regression).

### Multivariate Models of State Representations

For each subject and for each visual object independently, we trained a lasso-regularized logistic regression model to recognize instantaneous spatial patterns of MEG elicited by direct visual presentations of the object. The lasso penalty encouraged sparsity and tended to select occipital and posterior temporal sensors as useful features (Figure S2). Data used to train the regression models were taken from a secondary task in which the objects were presented multiple times in random order (see Experimental Procedures for details). Based on findings from our previous work (Kurth-Nelson et al., 2015), all models were trained only on MEG data recorded 200 ms after visual object onset.

**Figure 2:**
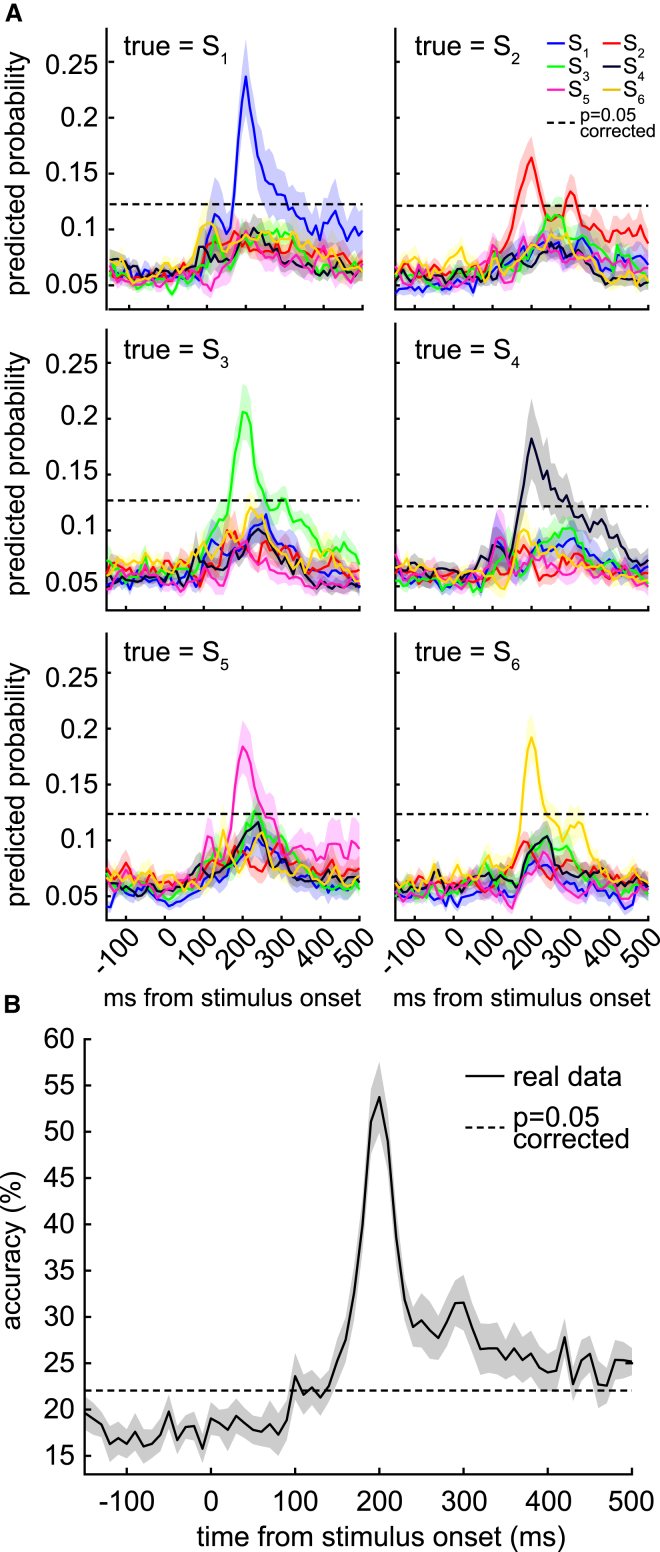
Lasso Logistic Regression Models Trained on Direct Presentations of Visual Objects For each participant, a separate regression model was trained to recognize each visual object. (A) We used leave-one-out cross-validation to test the generalizability of the learned models. These plots show the average probabilities output by the six models in cross-validation, with each panel corresponding to a different true state and each colored line showing the output of a different model. Because the models were trained only at 200 ms, correct prediction had a briefer peak than in other studies where classifiers were both trained and tested at every time point (e.g., Kurth-Nelson et al., 2015). (B) Prediction accuracy could be estimated by treating the index of the model with highest probability output as the predicted object. In cross-validation, the prediction accuracy reached 53.7% ± 3.8%, where chance was 16.7%. Dashed lines show 95% of empirical null distribution obtained by shuffling state labels. Shading indicates SEM.

Models were cross-validated on training data to confirm that they captured essential object-related features in the MEG signal. When models trained to recognize object *k* were tested on left-out data whose true class was *k*, the predicted probability reached 0.19 ± 0.02, peaking at 200 ms post stimulus onset. When models trained to recognize object *k* were tested on left-out data whose true class was not *k*, the predicted probability reached 0.08 ± 0.003 (Figure 2A). To ascertain that the models correctly decoded the objects, we also performed the same analysis 100 times with randomly shuffled state labels. In each shuffle, we used as a maximal statistic the maximum predicted probability over states and over time, thereby conservatively controlling for multiple comparisons. This yielded a set of 100 maximal statistics, and we used the 95th percentile of that set as a p = 0.05 significance threshold (dashed line in Figure 2A). All models’ actual predicted probabilities exceeded this threshold when the true stimulus was the same as what they had been trained to detect.

To test the specificity of the models, we analyzed prediction accuracies. The set of models together could be treated as making a categorical prediction about the class of the left-out data, by identifying the model with highest output probability on left-out data. The cross-validated prediction accuracy reached up to 53.7% ± 3.8%, where chance was 16.7% (Figure 2B). We also performed a similar shuffling procedure as above, using the maximum accuracy over time as the maximal statistic, and found that the true classification accuracy exceeded 100/100 shuffles.

### Sequences in Decoded Object Representations

**Figure 3:**
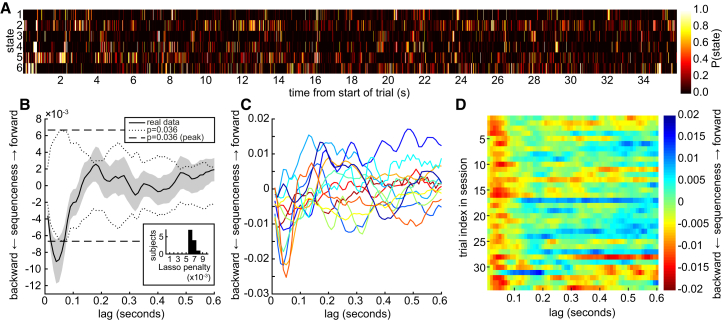
Decoded State Representations follow Reverse Sequences through the Maze (A) Probability time series output from the six regression models on a single example trial (one of 603 trials analyzed). Time zero corresponds to the start of the planning phase of the trial. We were interested in whether these time series contained any sequences following the transition structure of the task. (B) The y axis is the degree to which a neural representation of a state tended to be followed by (positive *y*) or preceded by (negative *y*) a neural representation of a successive state in the task (cf. Figure 1A), a measure we call sequenceness. This was quantified independently at all possible state-to-state lags (x axis). There was strong evidence for reverse sequenceness at around 40 ms of state-to-state lag. This effect was significant by parametric mixed intercept model (p = 0.0015 at 40 ms, not corrected for multiple lags) and by a non-parametric test based on shuffling the state identities. Dotted line shows non-parametric p = 0.036 threshold independently at each lag. Dashed line shows the peak of shuffles over all lags, which corrects for multiple lags, and was exceeded by the real data from 20 to 70 ms of lag. Inset: the lasso penalty for logistic regression models was selected by leave-one-out cross-validation over subjects to prevent over-fitting. Histogram shows distribution of lasso penalties across subjects. (C) 12/12 participants had reverse sequenceness at some time between 20 and 70 ms. Each line is an individual participant’s sequenceness plotted for all lags. Each participant is shown in the same color as in Figure 1. Individual data are shown at the group mode lasso penalty, 0.006. (D) The sequence effect was stable over trials within session (averaged over participants). Trials beyond 34 are not shown because few sessions exceeded 34 trials.

We applied the trained regression models to MEG data collected in the planning phase of each trial. As already described, this planning phase was a period that varied in duration from 2 to 60 s (cf. Figure 1D for distribution of times) during which no visual object was onscreen. Each 10 ms time bin of these data was independently input to each of the six regression models, yielding six time series of probabilities for each trial (example trial shown in Figure 3A). The probability in time bin *t* from model *k* quantified the degree to which the spatial pattern of MEG activity at time *t* resembled the evoked neural response to visual object *k*.

We next asked whether these time series contained sequences that followed possible paths in the behavioral task based upon a measure we refer to as “sequenceness.” For example, in the task there was a potential transition from state S_1_ to state S_2_. Sequenceness quantified whether a decoded neural representation of state S_1_ was likely to be followed by a decoded representation of state S_2_ or, in case of reverse sequences, whether S_2_ would be followed by S_1_. We operationalized sequenceness using a cross-correlation measure (see Experimental Procedures for details).

Sequenceness could either be forward (e.g., a representation of state S_2_ followed that of state S_1_) or reverse (e.g., S_2_ preceded S_1_). We observed a peak in reverse sequenceness at 40 ms of lag (Figure 3B). This signifies that if a neural representation of state *k* was active at time *t*, then a representation of one or both of the states with transitions to *k* was active around time *t* + 40 ms. We tested whether sequenceness at 40 ms lag was significantly different than zero using a multilevel model with a single fixed intercept term and one random intercept term for each participant. The fixed intercept term was estimated at −8.3 × 10^−3^ (p = 0.0015, two tailed). Next, in order to avoid relying on the assumptions of this model, we used a nonparametric method, shuffling the state identities 28 times to generate a null distribution of sequenceness (see Experimental Procedures for details). By taking the peak of the absolute value of each shuffle across all possible lags, and then taking the maximum of these peaks across shuffles, we obtained a conservative two-tailed significance threshold at approximately p = 1/28 ≈ 0.036. The real data exceeded this threshold from 20 to 70 ms of lag. We note that although the relatively small sample size in our study warrants caution, the consistency of sequenceness between participants (Figure 3C) was both striking and reassuring.

**Figure 4:**
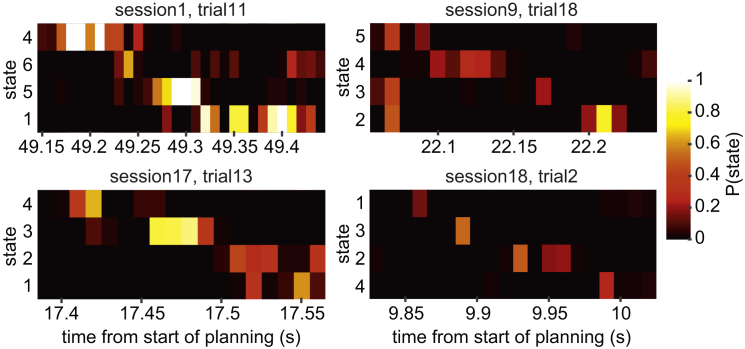
Example Sequence Events Time progresses rightward along the x axis. Each row depicts the probability outputs of one regression model, and the rows are sorted top to bottom to follow the reverse order of transitions in the task. Each example is from a different participant. We manually selected examples on the basis of looking good, so they should not be taken as statistically meaningful.

The logistic regression models had a free parameter defining the amount of regularization. To ensure the results were not an artifact of selecting a particular parameter value, the data shown in Figure 3B, and used for both parametric and non-parametric statistics described above, were obtained by cross-validating over this parameter. The parameter used to calculate sequenceness for each subject was the value that gave the strongest average sequenceness in the other 11 participants. The best value of the parameter was relatively consistent between cross-validation folds (Figure 3B, inset). Finally, the reverse sequenceness effect was stable across trials within each session (Figure 3D). Examples of individual sequence events, each lasting on the order of 100–200 ms, are shown in Figure 4.

### Length of Sequences

**Figure 5:**
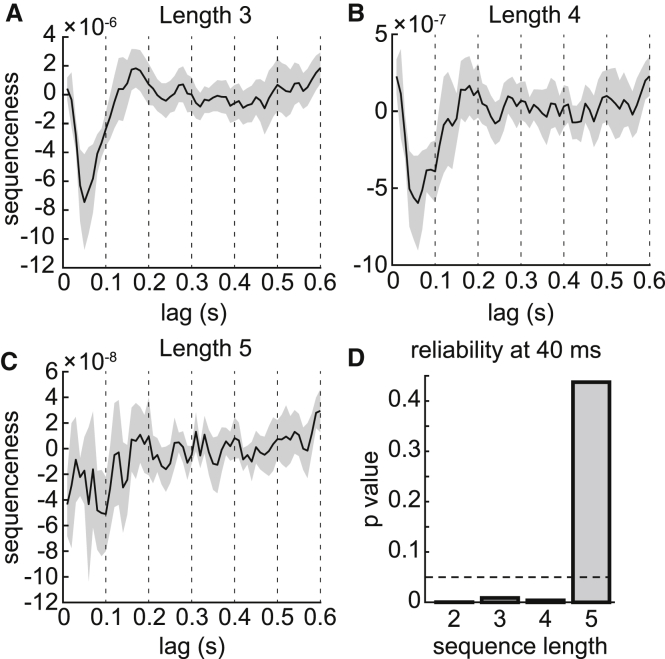
Sequences Are Reliable up to Length 4, but Not Length 5 (A–C) The sequenceness measure shown in Figure 3B could be generalized to longer sequences. These plots show the degree to which decoded state representations appeared in consecutive sequences of length 3 (A), 4 (B), or 5 (C). (D) The un-normalized inner products used to detect higher-order sequences did not bear direct comparison between different sequence length, but could be compared through their reliabilities, expressed as p values of the mixed intercept model described in the main text. Length 3 and length 4 sequences were nearly as reliable as the length 2 sequences shown in Figure 3, but no length 5 sequences were detectable at 40 ms lag. (A trend toward length 5 reverse sequenceness appeared at a slower state-to-state lag of 100 ms but did not reach significance.) A dashed line is drawn at p = 0.05 for reference.

We next asked whether the reverse sequenceness effect was driven by pairs of object representations appearing in isolated sequences (i.e., length 2 sequences) or by longer contiguous sequences. We explored sequences of length 3, 4, and 5. A length 3 reverse sequence would occur if, for example, state S_6_ had high probability at time t, state S_5_ had high probability at time t + 40 ms, and state S_4_ had high probability at time t + 80 ms. The length *n* sequenceness measure we used was a generalization of the cross-correlation measure used to detect length 2 sequences (see Supplemental Experimental Procedures for details). Because this method does not allow direct comparison of effect magnitude between different sequence lengths, we compared effect reliability using the same multilevel model described above. The fixed effect intercept term, which quantifies overall sequenceness, was reliably different from zero at 40 ms state-to-state lag for length 3 (p = 0.009) and length 4 (p = 0.004) sequences. The intercept term was not different from zero for length 5 sequences (p = 0.4). We conclude that spontaneous state representations tended to occur as fast sequences of up to four consecutive states (Figure 5).

Given that the sequence effect was strongest at a state-to-state lag of 40 ms, we estimated that an entire 4-state sequence, consisting of three transitions, lasted on the order of 120 ms. As in most rodent studies (e.g., Diba and Buzsáki, 2007, Dragoi and Tonegawa, 2011, Gupta et al., 2010, Ji and Wilson, 2007), these sequences were compressed in time relative to real experience. The most visually striking marker of state change during move execution was the visual cross-fade between objects, which took 350 ms. Relative to this, sequences were temporally compressed by a factor of 9. Meanwhile, the duration of an entire state transition, including the time to display reward information, varied from approximately 1 to 4 s. Relative to this benchmark, sequences were temporally compressed by a factor of ∼25–100.

### Negative Results

Between participants, there was no significant relationship between planning time and earnings (p = 0.3 by regression on subject means), between planning time and sequenceness (p = 0.5 by regression on subject means), or between earnings and sequenceness (p = 0.2 by regression on subject means). We note that the absence of evidence in inter-individual differences should be interpreted cautiously, as the small sample size of the study was ill-suited to detect such differences.

Finally, we asked whether we could detect any trial-by-trial relationship between neural sequences and behavior. Across trials, we regressed the magnitude of sequenceness against the actual earnings and found no effect (p = 0.83 by linear mixed effects). We also regressed the magnitude of sequenceness against planning time on the same trial and found no effect (p = 0.27 by linear mixed effects).

As a more specific test, we asked whether the specific sequences encoded in the MEG data on a trial were predictive of the moves a subject would actually make on that trial. To test this, we ran a similar analysis to the main sequence analysis, but looking at the cross-correlation between individual pairs of states at 40 ms lag. For each state pair, we again subtracted reverse (S_j_→S_i_) from forward (S_i_→S_j_) cross-correlations. This yielded a magnitude of “sequenceness” for each individual pair (Figure S5). At the group level, there was no evidence for differences between pairs (one-way ANOVA, F(11,132) = 0.92, p = 0.52), as would be expected because the mapping from state to visual object was randomized between participants. We then tested whether trial-by-trial variability in these individual pairs related to behavior. On each trial, participants chose a sequence of four moves, or four *i*→*j* tuples. We tested whether the magnitude of sequenceness for individual tuples in the MEG data was greater for chosen tuples than unchosen tuples and found no effect (p = 0.85 by mixed intercept model). We also repeated the same analysis but restricted to the first chosen move in each trial; again, there was no difference between neural sequenceness of chosen tuples versus unchosen tuples (p = 0.15). Finally, we evaluated whether the magnitude of sequenceness for individual tuples in the MEG data was greater for chosen tuples than unchosen tuples on the previous trial and again found no effect when analyzing all four moves (p = 0.62) or only the first chosen move (p = 0.42).

## Discussion

We show that spontaneous MEG activity plays out fast sequences of state representations, after participants have learned a non-spatial navigation task based on one-way connections between these states. These sequences formed trajectories of up to four states that progressed backward through the connections of the task. The sequences had a state-to-state lag of 40 ms, meaning that a whole trajectory lasted on the order of 120 ms. Although spontaneous sequences have been reliably observed in rodent spatial navigation experiments, this is the first report, to our knowledge, of such sequences in humans, as well as the first in a non-spatial task setting (although sequences in spatial tasks can be modulated by information about non-spatial context; Takahashi, 2015). These results suggest that fast non-local sequences may be a fundamental neural mechanism in decision making that is conserved across species and across problem domains.

### Non-spatial Sequences and Hippocampus

Although all previous observations of fast spontaneous sequences have used spatial tasks, most of the functions suggested for these sequences in learning and decision making apply to non-spatial as well as spatial settings. Accordingly, a role for fast neural state sequences has been hypothesized in a range of cognitive processes (Buzsáki and Moser, 2013, Carr et al., 2011, Pezzulo et al., 2014).

Tolman made the seminal suggestion that an agent should build abstract cognitive maps for non-spatial as well as spatial tasks (Tolman, 1948). A computational view has emerged that this is a role played by the hippocampus and associated brain structures in a wide range of cognitive problems (Allen et al., 2016, Collin et al., 2015, Eichenbaum and Cohen, 2014, Killian et al., 2012, Milivojevic and Doeller, 2013, Muller et al., 1996, O’Keefe and Nadel, 1978, Redish, 1999, Schapiro et al., 2012, Shapiro and Eichenbaum, 1999, Takahashi, 2013, Tavares et al., 2015). If so, the hippocampus should express sequences in non-spatial tasks as it does in spatial tasks.

However, although MEG contains signals of hippocampal origin (Dalal et al., 2013), and several studies have reported source localizing MEG activity to hippocampus (e.g., Cornwell et al., 2008, Guitart-Masip et al., 2013), these signals are relatively difficult to detect, rendering it unlikely that the sequences we recorded arose directly from hippocampus. First, a fall-off of magnetic field strength with the square of distance from neural sources ensures cortical activity dominates the MEG signal (Hämäläinen et al., 1993, Riggs et al., 2009, Stephen et al., 2005). Second, our classifiers were trained on evoked visual responses shortly after visual stimulus onset, which can be assumed to reflect most strongly activity arising in a cortical visual processing stream. Third, the sensors used by the regularized regression models were mostly occipital and posterior temporal sensors in a pattern different from that reported for putative hippocampal activity (Dalal et al., 2013, Guitart-Masip et al., 2013).

The fact that our classifiers were trained on visual evoked activity makes it most likely that the observed sequences corresponded to reactivation of visual representations. In humans, the pattern of cortical activity observed during direct experience with a sensory object is at least partially reinstated when the object is retrieved or remembered (Danker and Anderson, 2010, Kuhl and Chun, 2014, Nyberg et al., 2000, Polyn et al., 2005, Wimmer and Shohamy, 2012). This reactivation is implicated in model-based reasoning, and we have previously shown that it can be tracked at fast timescales with MEG (Kurth-Nelson et al., 2015).

Since hippocampus drives cortical representations during retrieval (e.g., Tanaka et al., 2014), it is possible that sequences generated in hippocampus (perhaps during sharp-wave ripples [SWRs]) might drive sequences of cortical activity detected by our classifiers. This would be consistent with a coupling observed between hippocampus and cortex during SWRs (Siapas and Wilson, 1998, Sirota et al., 2003, Wierzynski et al., 2009) and the coordination of hippocampal and cortical sequences (Ji and Wilson, 2007). However, this remains speculative, and our data provide no direct evidence of an upstream role for hippocampus. It is entirely possible that the sequences we observed originated from intrinsic cortical dynamics, in keeping with prior observations that spontaneous space-related sequences occur in a variety of cortical areas in rats and primates (Euston et al., 2007, Hoffman and McNaughton, 2002, Ji and Wilson, 2007).

### Forward versus Reverse Sequences

Our main analysis was based on subtracting reverse from forward sequenceness. At most latencies, there was no difference between the two. However, at a lag of around 40 ms, there was much stronger expression of reverse compared to forward sequenceness. In principle, this effect at 40 ms could reflect either an increase in reverse sequences or a reduction in forward sequences, relative to baseline. However, we think the latter unlikely because it would imply a consistent positive amount of forward sequenceness at all other latencies.

Sequences within hippocampal SWRs in rats, which occur when the animal is pausing or resting, have been observed in both forward and reverse order (Diba and Buzsáki, 2007, Foster and Wilson, 2006, Gupta et al., 2010, Nádasdy et al., 1999, Skaggs and McNaughton, 1996). Theta sequences, which occur during active behavior, are nearly always forward (Foster and Wilson, 2007, Gupta et al., 2012, Johnson and Redish, 2007, Wikenheiser and Redish, 2013, Wikenheiser and Redish, 2015). This raises the question of the behavioral state of our subjects during the planning period of up to 60 s when sequences were observed. Given the relatively long time permitted for planning (up to 60 s) in our task, and the high attentional demands at other times within the task, it is entirely plausible that sequence events seen in our study corresponded to moments when people were pausing from actively calculating moves.

Another interesting question concerns whether neural sequences are “local” or “remote,” meaning whether or not they represent a trajectory that includes the current state of the agent. In rodents, theta sequences contain (but do not necessarily begin or end at) the current location of the animal (Gupta et al., 2012, Johnson and Redish, 2007, Wikenheiser and Redish, 2015). By contrast, SWR sequences sometimes include the current location of the animal (Diba and Buzsáki, 2007, Foster and Wilson, 2006) but also encode trajectories that do not include the current location of the animal (Davidson et al., 2009, Gupta et al., 2010, Karlsson and Frank, 2009). In our MEG data, sequences containing the state pair of the participant’s first move on the trial were no more common than sequences not containing this pair, suggesting that sequences were as likely to be initiated remotely as locally.

### Length of Sequences

Hippocampal sequence events in rodents last on the order of 50–200 ms, whether they occur in theta (Wikenheiser and Redish, 2015) or in SWRs (Diba and Buzsáki, 2007). This is similar to the 120 ms length we estimate for a four-state sequence event in our data. In rodent navigation experiments, states are defined continuously in space rather than discretely as in our maze, which does not admit a direct comparison with the number of states visited.

### Retrospection versus Prospection

In the rodent literature, a number of functions are imputed in common to both forward and reverse sequences. This is particularly true of retrospective functions including assigning credit to recent experience (Foster and Wilson, 2006), consolidating memory (Carr et al., 2011), or active memory maintenance (Káli and Dayan, 2004). Consistent with a role in learning and memory, new experiences boost the frequency of SWRs (Eschenko et al., 2008) and boost coordination of neuronal activity within these SWRs (Cheng and Frank, 2008), while disruption of SWRs harms memory performance (Girardeau et al., 2009, Jadhav et al., 2012). The complexity of our task raises the possibility that consolidating knowledge of task structure was ongoing during performance, and the sequences we observed in MEG might play a role in this process.

Sequences ascribed prospective functions have most often been of a forward variety (Johnson and Redish, 2007, van der Meer and Redish, 2009, van der Meer et al., 2010, Pfeiffer and Foster, 2013, Wikenheiser and Redish, 2015). The reverse order of our sequences, along with the fact that they were not more likely than chance to contain the subsequently chosen state sequence, might argue against a prospective function. However, reverse sequences with a possibly prospective function were observed in Ólafsdóttir et al. (2015), and some rodent studies postulating a prospective function for sequences observed no simple relationship between neural sequences and upcoming behavior (e.g., Johnson and Redish, 2007). It is perfectly possible to plan backward instead of forward (e.g., LaValle, 2006), and it would be premature to conclude that the sequences we observed have purely retrospective functions. One interesting future test would be to collect MEG data during times when participants do not have access to information they need to plan an upcoming action. If sequences are in fact used for online planning, we would expect they should not occur when participants are not actively engaged in planning.

Intermediate between retrospective and prospective accounts, the observed sequences might also play a role in working memory. Even after training, participants reported that recalling the transitions was effortful. Fast neural sequences could reflect a form of online working memory, interfacing between long-term memory and planning mechanisms. Another possible intermediate function is offline planning (Shohamy and Daw, 2015, Sutton, 1991), where values of paths or sub-paths might be calculated and stored in anticipation of future use.

### Conclusions

Our results highlight the power of multivariate analysis of MEG data to trace the trajectories of fast-evolving neural representations in humans. In addition to being non-invasive, multivariate MEG has an added benefit of tracking representations that may be distributed across wide cortical areas. Applying this methodology has enabled us to provide the first evidence for fast spontaneous sequences of state representations in the human brain. Additionally the findings demonstrate this neural motif outside the spatial domain. Thus, fast sequences appear to be a fundamental principle of neural computation in a range of cognitive domains.

## Experimental Procedures

### Participants

12 adults aged 18–31 participated in the experiment, recruited from the UCL Institute of Cognitive Neuroscience subject pool and from a mailing list for MSc students. Six were female and two were left-handed. All participants had normal or corrected-to-normal vision and had no history of psychiatric or neurological disorders. Eight of the 12 participants underwent two scanning sessions, for a total of 20 recorded sessions. Two of these sessions were excluded before the start of analysis owing to large artifacts, leaving 18 analyzed sessions. All participants provided written informed consent and consent to publish prior to start of the experiment, which was approved by the Research Ethics Committee at University College London (UK), under ethics number 1825/005.

### Task

In the MEG scanner, participants performed a 6-state sequential reasoning task inspired by Huys et al., 2012, Huys et al., 2015 but designed with the additional criterion of encouraging mental representation of the visual objects that identified each state. The task was implemented in MATLAB (MathWorks) using Cogent (Wellcome Trust Centre for Neuroimaging, University College London). Each trial began with the participant being placed at a randomly selected state within the maze. From this state, they were permitted four sequential moves with the instructed aim of maximizing their earnings. From each state, a move constituted one of two possible choices, called “up” and “down” (so-called for simplicity of button pressing, although there was no meaningful spatial relationship between the states). Each of these choices deterministically led to a different next state. Only one state was ever viewed at a time, and participants never saw a bird’s-eye view of the maze.

Each state provided a monetary outcome of between −5 and +5 pence. The reward for each state drifted independently at random by −1, 0, or +1 pence on each trial. Upon reaching a state, the state’s current reward value was added to the participant’s running total for that trial. This running total was also displayed on the screen while moves were being executed. Finally, in each trial, two randomly selected states were designated as “neg” states. When a neg state was reached, first its reward value was added to the running total for the trial as usual, but then the sign of the running total for the trial was flipped (e.g., −9 became +9 and vice versa). The identities of the neg states were signaled in text at the beginning of each trial. In many trials, the optimal strategy involved the use of one or two neg states. Two neg states could be used within a trial to reach a positive total reward, or a single neg state could be used in conjunction with negative state reward.

On each trial, participants were first shown in text the names of the starting state and the two neg states and allowed up to 60 s to plan. After the end of the planning period, participants were faced with a blank screen upon which they could pre-enter their chosen sequence of four moves. They were allowed up to 3 s to enter the first move and 1 s for each of the last three moves. As they pre-entered each move, a corresponding up or down arrow appeared on the screen for confirmation, but no visual objects were shown. After pre-entering all four moves, the visual object corresponding to the starting state of this trial appeared. Participants were then required to repeat the sequence of moves they had pre-entered. As they executed each move, the visual object shown on the screen changed to reflect the corresponding state transition. Up to 10 s was permitted to execute each move. Executing each move was followed by 350 ms of animated cross-fade transition between visual objects, followed by 500 ms pause, followed by the current reward amount of the new state displayed for 1,000 ms, followed by the total trial earnings being updated and displayed for 1,000 ms, followed by a neg and corresponding change to total trial earnings, if any, being displayed for 1,000 ms. If this was the final move of the trial, the final reward for the trial was then displayed for 3,000 ms; otherwise, the next move could be entered.

The task design was motivated by a wish to encourage participants to learn and use the transition structure of the task, instead of relying on simple choice strategies like repeating reinforced actions. We reasoned that engaging participants with the transition structure would afford us the best chance of detecting neural sequences reflecting this structure. Two other features of the task design were also intended to meet this purpose. First, trials were generated such that simple choice strategies would yield much lower payouts than optimal planning strategies (see Supplemental Experimental Procedures for more detail). Second, pre-entry of the four sequential moves on each trial was made in the absence of feedback about the consequences of those moves until all four had been entered. This meant that participants had to anticipate where move *m* would lead in order to make a good decision on move *m* + 1.

Each of the six states in the maze was a unique visual object. For each participant, the six objects were drawn randomly from a set of ten objects (bird, bread, cat, chair, garlic, hammer, hand, horn, tree, water), and the six chosen objects were randomly assigned to the six states of the maze.

After the 6-state reasoning task, participants completed a secondary task while still in the scanner. This task was designed to elicit neural representations of known stimuli, which could be used to train classification models. In this secondary task, the name of a visual object appeared in text for a variable duration of 1,500 to 3,000 ms, followed immediately by the visual object itself. On 20% of trials, the object was upside-down. To maintain attention, participants were instructed to press one button if the object was correct-side-up, and a different button if it was upside-down. Once the participant pressed a button, the object was replaced with a green fixation cross if the response was correct and a red cross if the response was incorrect. This was followed by a variable length inter-trial interval of 700 to 1,700 ms. Each session included 125 trials of the secondary task, with approximately 16 correct side-up presentations of each visual object. Only correct-side-up presentations were used for classifier training. The trial order was randomized for each participant. Per participant, the visual objects used were the same six objects used in the main task.

### Behavior Analysis

We fit four models to explain participants’ choices. “Plan” used a full-depth tree search to calculate the value of each of the 16 possible sequences of four moves on each trial. This model represented optimal behavior on the task. “Qfirst” used no knowledge of the task’s structure but learned Q-values for the 12 available state-action pairs based only on the first move in each trial. “Qall” also learned Q-values for the 12 available state-action pairs but exploited knowledge of the task structure to evaluate actions and update Q-values for all four moves in each trial. “Greedy” also used knowledge of the task structure but, rather than learning Q-values, selected each move in sequence by maximizing expected return locally from that move. Details of models and model comparison are given in the Supplemental Experimental Procedures.

### MEG Acquisition and Pre-processing

MEG was recorded continuously at 600 samples/second using a whole-head 275-channel axial gradiometer system (CTF Omega, VSM MedTech), while participants sat upright inside the scanner. Participants made responses on three buttons (called “up,” “down,” and “advance”) of a button box using the fingers they found most comfortable.

The data were resampled from 600 Hz to 100 Hz to conserve processing time and improve signal to noise ratio. Thus, data samples used for analysis were spaced every 10 ms. All data were then high-pass filtered at 0.5 Hz using a first-order IIR filter to remove slow drift. All analyses were performed directly on the filtered, cleaned MEG signal, consisting of a length 134 vector of samples every 10 ms, in units of femtotesla.

### Multivariate MEG Analysis

Lasso-regularized logistic regression models were trained on MEG data elicited by direct presentations of the visual objects. These presentations were taken from the secondary task that succeeded the 6-state reasoning task in the scanner, specifically the data 200 ms following stimulus onset. This 200 ms time point was selected based on observations from our previous work (Kurth-Nelson et al., 2015), which showed that when object representations are retrieved, the reinstated spatial pattern used in value reasoning is most similar to the pattern observed 200 ms after onset of direct object presentation. This constitutes the only available information to our knowledge about which of the time series of patterns evoked by direct object experience might be reinstated during decision making.

Models were verified on training data through cross-validation. In each cross-validation fold, we partitioned the data randomly, under constraints that ensured balanced classes for training: (1) each object had at least one left-out trial, (2) each object had the same number of left-in trials, and (3) the number of left-out trials was minimized subject to the other two constraints being satisfied.

A trained model k consisted of a single vector β_k_ with length 135: slope coefficients for each of the 134 sensors together with an intercept coefficient. We used these trained models to make predictions as to whether unlabeled MEG data corresponded to a neural representation of visual object *k*. Each time point was treated independently. At each time point in the unlabeled data, the data vector over sensors was multiplied by β_*k*_ and transformed by a sigmoid to obtain a predicted probability for visual object *k*. This procedure yielded six probabilities at each time point; so for each trial we obtained a matrix X with six columns and as many rows as time bins in the trial.

### Sequenceness Measure

Inspired by cross-correlation measures used in the analysis of spike data from rodent hippocampus (e.g., Hoffman and McNaughton, 2002), we used the matrix X to calculate a “sequenceness” measure. Sequenceness operationalized the degree to which decoded MEG activity tended to follow the transition matrix of the task systematically in either a forward or reverse direction. For example, in the task, state S_1_ admitted a transition to state S_2_ (but not S_3_, S_4_, or S_6_; cf. Figure 1A). If X contained forward sequences, then the decoded probability of S_1_ at time T should be correlated with the decoded probability of S_2_ at time T + *t*, where *t* defines a lag between neural state representations. Meanwhile, reverse sequences would be expected to support the opposite decoding order.

The sequenceness measure was calculated independently for each possible lag t. We first multiplied X by the transition matrix of the task (assuming equal probabilities for the two possible actions from each state) to obtain X^F^ and then calculated the cross-correlation at varying time-lags between column *i* of X and column *i* of X^F^. This produced six correlations for each possible time-lag. These six correlations were averaged to obtain a single number representing the overall forward sequenceness at that time-lag. In parallel, we also multiplied X by the reverse transition matrix of the task to obtain X^R^ and again calculated the cross-correlation at varying time-lags between X and X^R^. This yielded the reverse sequenceness at each time-lag. We finally subtracted the reverse sequenceness from the forward sequenceness at each time-lag.

As a parametric test for the difference of sequence effect from zero, we estimated a mixed model with only intercept (and no slope) terms.

$$y_{i}=\beta +b_{k}+error_{i},$$

where *y*_*i*_ was the sequenceness on trial *i*, β was the fixed effect intercept, *k* was the participant on trial *i*, and *b*_*k*_ was the random effect intercept for participant *k*. *b*_*k*_ were assumed to be normally distributed with variance σ. The model was estimated using Matlab’s fitlme function.

We also protected the statistical inference using non-parametric permutation tests involving all possible ways of scrambling the six state labels. There were 30 unique permutations of the state labels up to symmetries in the transition matrix of the task. One of these was the identity permutation and one was the reverse-order permutation. For non-parametric significance testing, we therefore used the peak of the absolute value of the other 28 unique permutations as a threshold, at an approximate empirical two-tailed p value of 1/28 ≈ 0.036.

Although sample order is sometimes shuffled in rodent electrophysiology experiments, we found in simulations that the substantial autocorrelation in the MEG time series meant that shuffling sample order led to very high false positive rates (Figure S4). Shuffling state identity is a more conservative measure, which allowed us to reject the null hypothesis that the MEG time series had no relationship to the transition structure of the task.

Importantly, the statistical tests protect against the possibility of systematic dynamics in brain activity (for example, anterior to posterior sweeps of activity) leading to false detection of sequences. Any such activity not reflecting the transition structure of the task would, by definition, be unbiased in whether it appeared to progress forward or backward through the task transitions.

Up to 60 s of MEG data per trial were analyzed for sequenceness, potentially raising a question of whether false positives could arise through the sheer amount of data analyzed. However, this can be ruled out. The cross-correlation analysis does not report the total number of sequences, but the density of sequences per time, meaning that analyzing a longer period of MEG data would not inflate the sequenceness measure. Also, the permutation test used scales to the amount of data, since each permutation is calculated on the full data.

### Testing for Length-*n* Sequences

We also computed the extent to which neural sequences followed multiple consecutive steps with the same state-to-state lag, using a similar procedure as described above. Multiplying X by the task’s transition matrix twice, we obtained X^FF^. We then element-wise multiplied column *i* of X, column *i* of X^F^ shifted by lag *t*, and column *i* of X^FF^ shifted by lag 2*t* and summed the product. For each lag *t*, this yielded six numbers akin to cross-correlations except for being un-normalized. These six numbers were averaged to obtain a single number representing the overall propensity for two forward steps to occur consecutively (which we call “length 3” sequences because they consist of three states). An analogous procedure applied to reverse transitions. This process could be generalized to any number of steps. This method explicitly asks whether all states in a length-*n* sequence occur and is not sensitive to sequences that skip intermediate states.

All summary statistics are given as mean ± SE, unless otherwise stated. Median and IQR are given when the underlying distribution is substantially non-Gaussian.

## Author Contributions

Z.K.-N., P.D., and R.J.D. designed the study and prepared the manuscript. Z.K.-N. and M.E. acquired the data. Z.K.-N. and P.D. analyzed the data.

## Supplemental Information

Document S1. Supplemental Experimental Procedures and Figures S1–S5

Document S2. Article plus Supplemental Information
