# Supplementary material for: Fast Sequences of Non-spatial State Representations in Humans
Source: Neuron. 2016 Jul 6;91(1):194–204. doi: 10.1016/j.neuron.2016.05.028 (PMC4942698; doi:10.1016/j.neuron.2016.05.028)
Supplement: Document S1. Supplemental Experimental Procedures and Figures S1–S5 [file mmc1.pdf]

Neuron, Volume *91*

Supplemental Information

Fast Sequences of Non-spatial

State Representations in Humans

Zeb Kurth-Nelson, Marcos Economides, Raymond J. Dolan, and Peter Dayan

Supplemental Figures

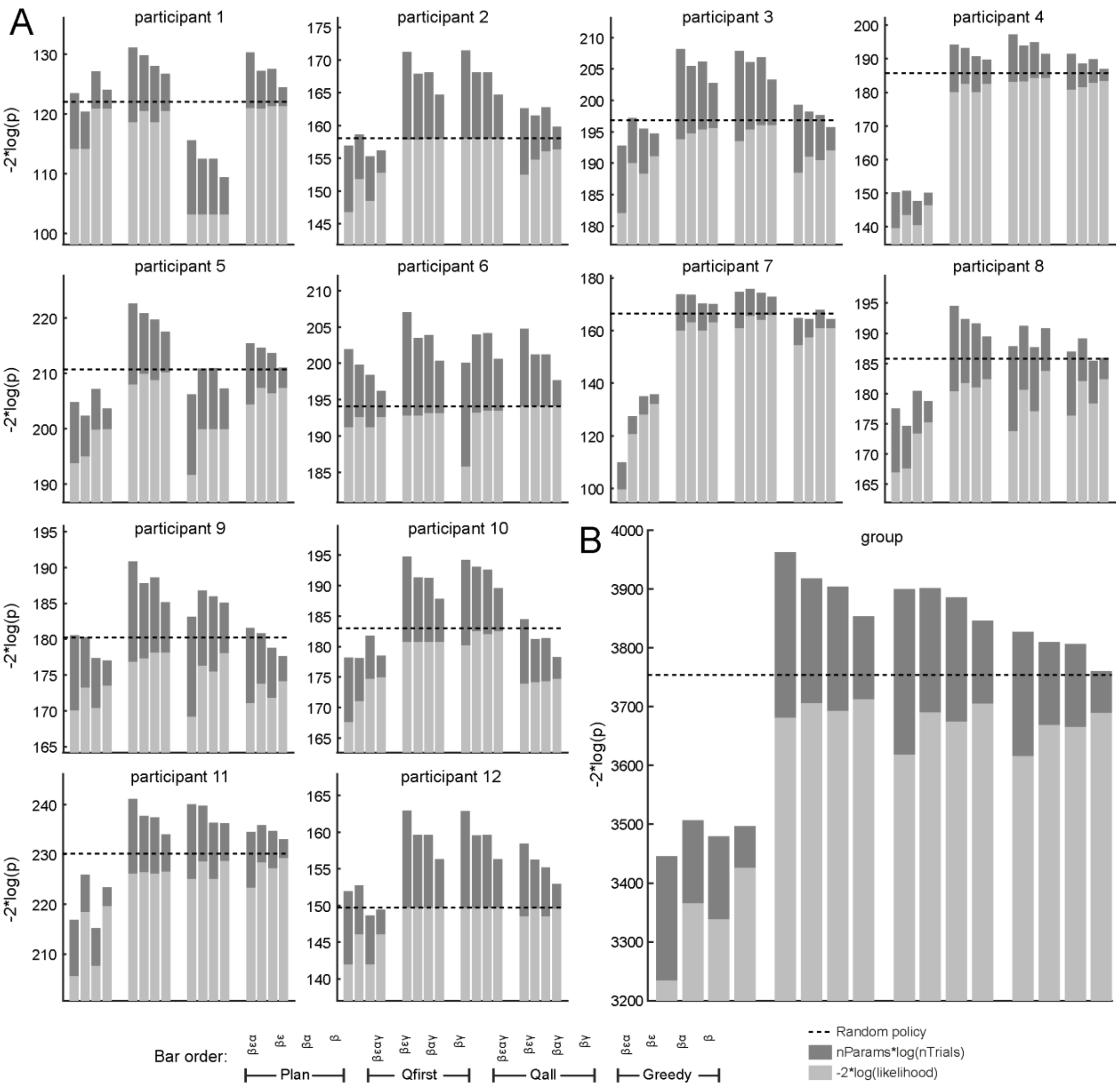

[Related to Figure 1]

Figure S1 - Behavioral model comparison. Four different models were fitted to behavioral choice data: "Plan", "Qfirst", "Qall" and "Greedy". Each model is described in the Experimental Procedures. Each model always had an inverse temperature parameter β, and the Q-learning models always had a discount rate parameter γ. There were two optional parameters in each model: a lapse rate ε and a learning rate α. Each combination of including or excluding ε and α was explored for each model. Light gray bars show two times the negative log of maximum likelihood. Dark gray bars show the BIC model complexity penalty, which depends on the number of parameters. The sum of these two quantities is an approximation of two times the total negative log evidence for each model. **A,** In 11/12 participants, the planning model outperformed the simple RL models. The order of the bars in each plot is the same as in Figure S2. Note that the evidence for the random policy depended on the number of trials completed, and so differed between participants. **B,** At the group level, the planning model was favored over the best simple RL model by 157 log units of evidence. The simple RL models did not perform better than chance, after taking into account the complexity penalty.

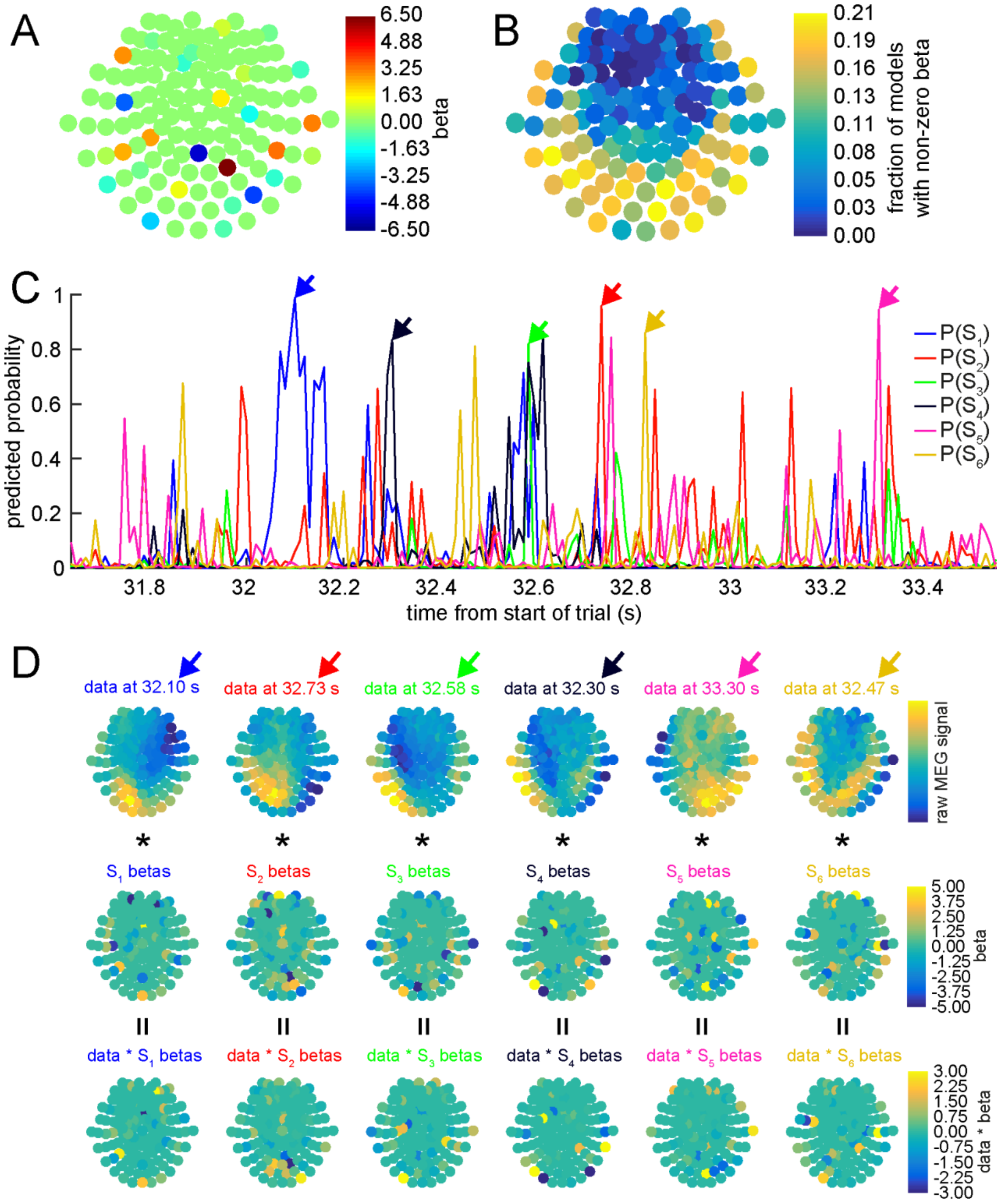

[Related to Figure 2]

Figure S2 – Decoding methods. For each session, six lasso-regularized logistic regression models were trained, one for each visual object in the primary task. **A,** The lasso penalty encouraged sparsity, so the majority of coefficients in each trained model were zero. One example trained model is shown here (intercept not shown). **B,** These models tended to select mostly occipital and temporal sensors. Of the 108 models used in analysis (18 sessions times 6 models), this plot shows the fraction where each sensor was non-zero. **C,** An example of the six probability time series output by the six regression models, over a short segment of time. Six time points, indicated by colored arrows, were selected for display in D. **D,** The top row shows the raw MEG data at the six time points indicated by arrows in C. The middle row shows the learned betas for the classifier whose output is high at this

time. The bottom row shows the element-wise product of the data at that time point with the illustrated classifier. The mean across sensors of these products, plus the intercept of the model, was the value *x* used to generate a probability through the transformation 1 / (1 + exp(-*x*)).

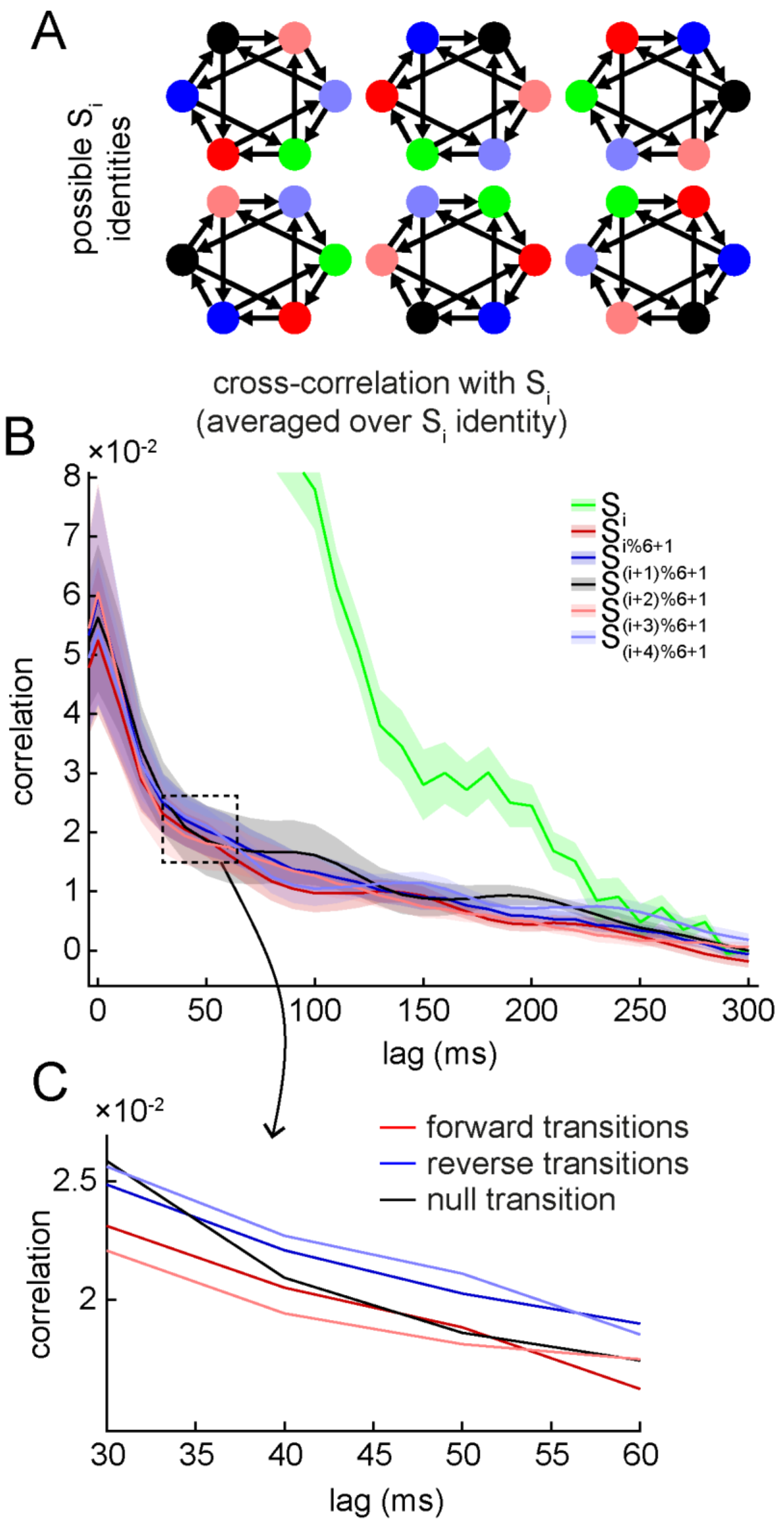

[Related to Figure 3]
Figure S3 – Autocorrelation in classifier outputs. Cross-correlations between pairs of states had a strong peak near zero lag due to autocorrelation in the underlying MEG signal. **A,** Symmetries over

state relationships, defining the color mapping used in B and C. **B,** Cross-correlations were calculated for each ordered pair of states, yielding 36 cross-correlations. These were collapsed by averaging over equivalent transitions (under the symmetries shown in A; for example, S_1->S_2 was equivalent to S_2->S_3), giving six cross-correlations, which are shown here. At 40 ms lag, the reverse transitions (blue and light blue) had stronger correlation than the forward transitions (red and light red). The reverse transitions also had stronger correlation than the null transitions (black), but this was not statistically significant. The identity transition (green) had strong autocorrelation. **C,** Magnification of lags from 30 to 60 ms. The difference between the blue traces and the red traces reflects the sequenceness effect shown in Figure 3 in the main text.

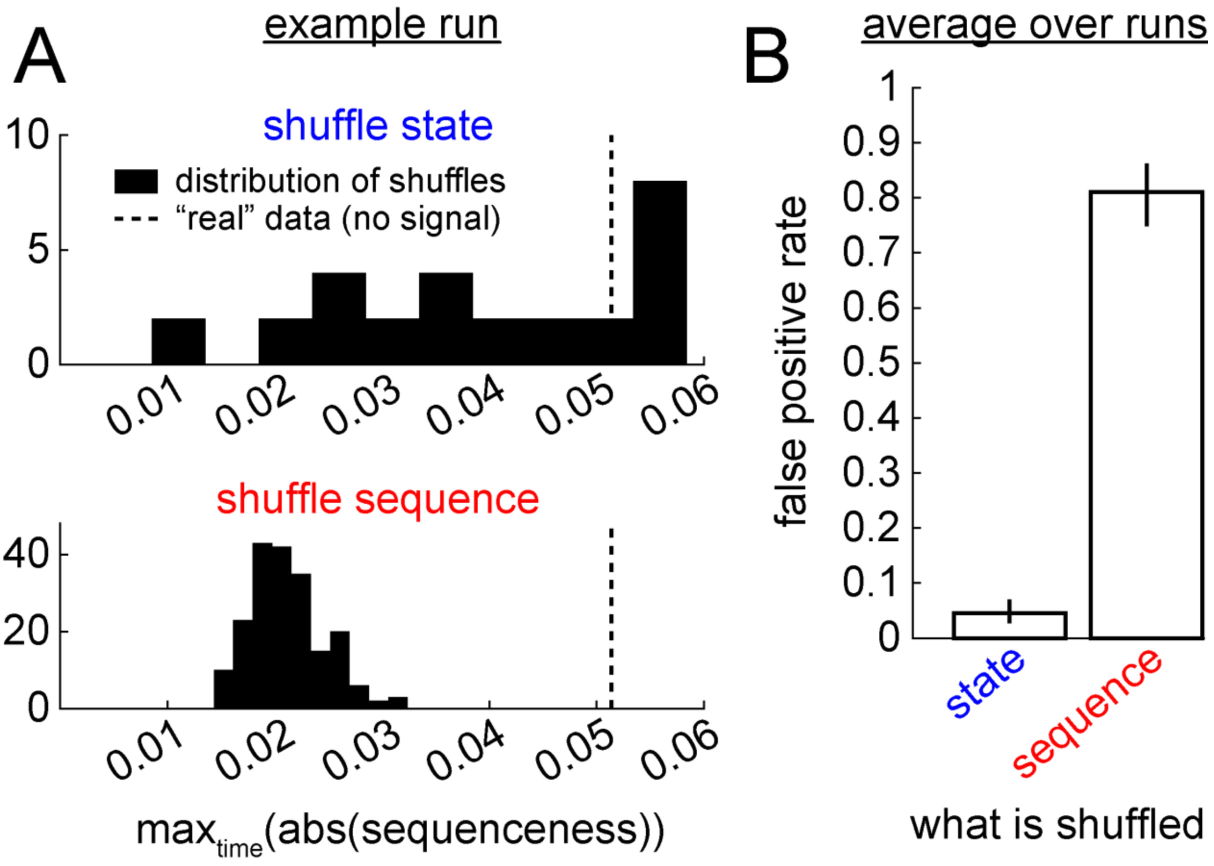

[Related to Figure 3]

Figure S4 - Shuffling sequence produces false positives. We simulated six autocorrelated time series of classifier predictions in which there was no true sequenceness. **A,** We applied our sequenceness analysis method to this synthetic data to obtain sequenceness for lags between 10 and 600 ms, in 10 ms increments. The maximum of the absolute value of these sequenceness measures over all lags is shown as a dashed line. We then applied the same state shuffling procedure used on our MEG data to obtain a null distribution of maximum statistics (black histogram, top panel). The "real" data did not exceed either the maximum of this distribution (the criterion we used on our MEG data), or the 95th percentile, consistent with the fact that there was no sequenceness in the simulated data. However, when we used a shuffling procedure that shuffled the temporal sequence of the data instead of shuffling state identities, the "real" data exceeded the maximum of the null distribution, because shuffling sequence creates an artificially narrow null distribution. **B,** We repeated the procedure in A 400 times for each shuffling method. The "real" data exceeded the null distribution of the state shuffling method in 4.5% (95% binomial CI [2.7%, 7.0%]) of the repetitions. However, despite there being no signal in the data, the "real" data exceeded the null distribution of the sequence shuffling method in 81% (95% binomial CI [75%, 86%]) of the repetitions.

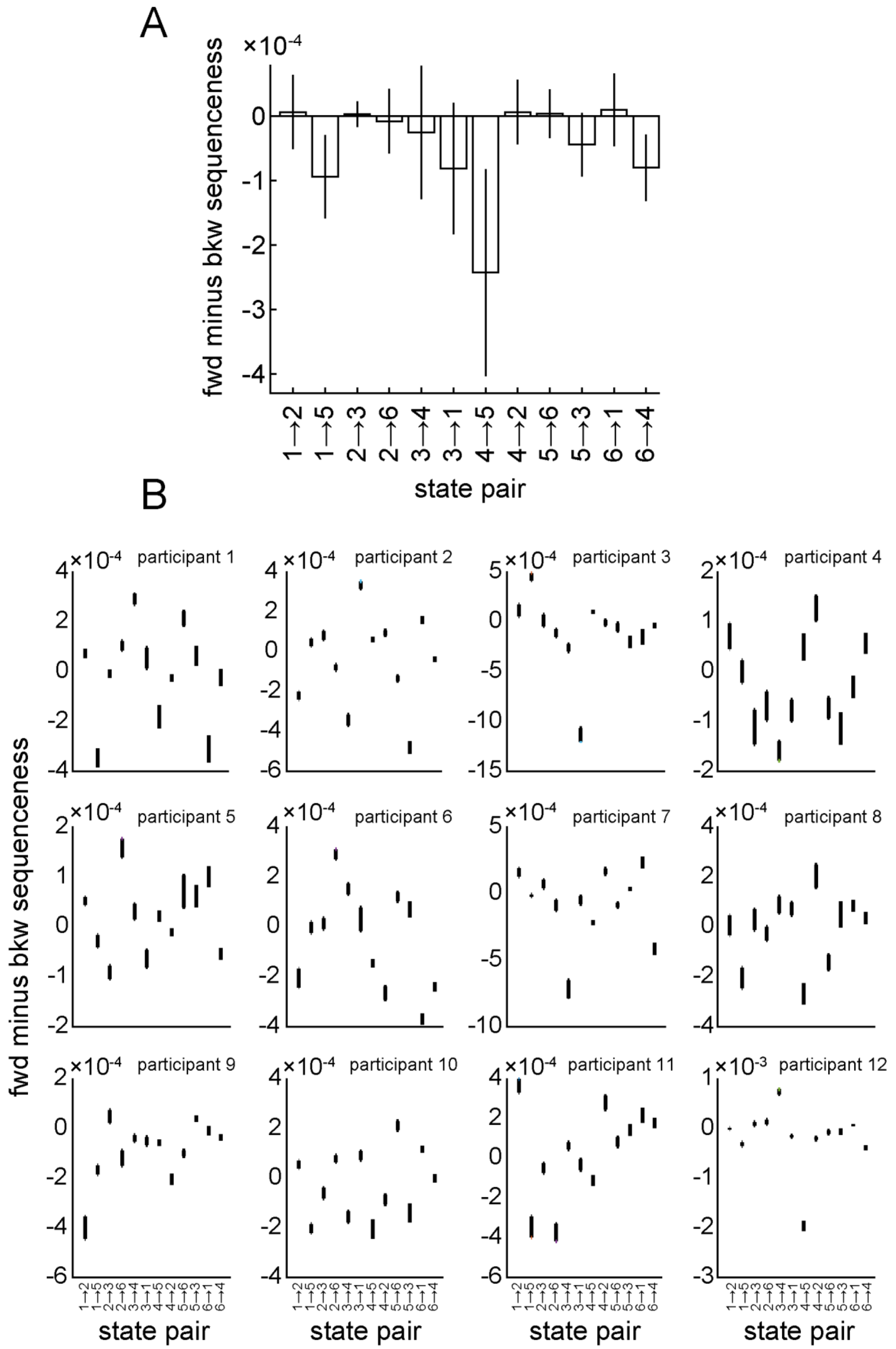

[Related to Figure 3]

Figure S5 - Sequenceness at 40 ms for each state pair. For each ordered pair of states having a transition between them in the task (shown on the x-axis of each panel), we subtracted the cross-correlation between these states at -40 ms from that at +40 ms. This subtracted cross-correlation is shown on the y-axis in all panels. **A,** In the group average, there was no reliable difference between state pairs by one-way ANOVA ($F(11,132)=0.92$, $p=0.52$). This was expected, given that the visual objects associated with the states were randomized across participants. **B,** In each individual participant, there was a significant difference between state pairs that was reliable across trials (ANOVA F-statistics ranged from 8 to 84).

Supplemental Experimental Procedures

Task

From a participant's point of view, each trial involved three phases: planning, move pre-entry, and move execution. During planning, participants saw in text the names of the starting state and the two neg states for that trial. Participants had up to 60 seconds to plan their moves, but could also press a button to enter their moves sooner. After the 60 seconds or this button press, there was a 1-second warning to signal the start of the move pre-entry phase. During move pre-entry, participants were required to enter their chosen sequence of four choices rapidly, while receiving no visual feedback except the corresponding arrows (up and down) appearing to reflect their move selections. If they took longer than 1 second per move, the trial was aborted with a fixed -10 pence loss. One percent of all trials was aborted due to time-outs.

Next, during move execution participants were required to play out the sequence of four moves they selected during pre-entry. On each move, the button corresponding to the unchosen option (up or down) was deactivated, so there was no possibility of deviating from the pre-entered sequence. During execution, the visual objects corresponding to each state were shown as the participant moved from state to state. The transitions between objects were visually cross-faded with a blend ratio that changed linearly from 100%/0% to 0%/100% over 350 ms. The current reward values of each object were also shown, and the cumulative reward total for the trial was updated as each new state was reached. If a state was neg, the cumulative reward total would first reflect the addition of the state's reward value, and then the text "neg" would appear and the displayed cumulative reward total would flip its sign.

The transition structure of the maze was identical for all subjects. The assignment of "up" and "down" to each transition was randomized between subjects. The pseudo-random sequence of rewards, starting states, and negative states was also identical for all sessions (although the number of trials completed varied between sessions due to the self-paced nature of the task) and was optimized using a genetic algorithm. The primary goal of optimization was to arrange it that on nearly half of trials, optimal and greedy choices differed for the first choice in the sequence, where greedy choice means selecting the highest-value next state. (If this target had been more than a half, subjects could have employed the shortcut of doing the opposite of the greedy strategy.) A secondary goal of optimization was avoiding degenerate cases such as repeating the same starting conditions.

At the end of all training and scanning sessions, participants received their earnings on the task, which ranged from £-0.60 to £1.76, plus £10 for each day of behavioral training and £15 for each day of scanning. The amount of money that could be earned varied substantially from trial to trial, so in the paper per-trial earnings are reported as $E - E_{rand}$, where E was the actual earnings on the trial, E_{rand} was the expected earnings under random play on this trial (both in units of pence).

Behavioral training

Participants received either two or three days of training on the task structure before scanning. Training progressed until a participant reached 100% performance on the final set of quizzes used for training. Training started by introducing the six visual objects and quizzing participants about single transitions (e.g., if you are in "tree" and you choose "down", which state will you arrive at?). Quizzes were then extended to two-move sequences and four-move sequences. Finally, rewards were introduced, and participants were quizzed about how much money they would earn by taking specified sequences of four moves.

The visual objects and transition structure used during training were preserved for each subject into the actual experiment, but the rewards used during training were independent of the rewards used in

the actual experiment. All training was automated in MATLAB/Cogent to minimize experimenter interaction.

Behavioral models

We fit four models of choice behavior. "Plan" used an optimal full-depth tree search to calculate the value of each of the 16 possible sequences of four moves on a trial. This planning depended on tracking the drifting rewards associated with each state. The model allowed for gradual learning about the drifting rewards, following a learning rate (α). Each time the current reward R_i for state *i* was revealed, the tracked reward ρ_i was updated:

$$\rho_i \leftarrow \rho_i + \alpha \cdot (R_i - \rho_i)$$

The values, V, of each of the 16 possible sequences of four moves were calculated optimally and transformed to choice probabilities with a sigmoidal link function parameterized by inverse temperature (β), and lapse rate (ε):

$$P_i = \varepsilon + (1 - 16\varepsilon) \cdot \frac{e^{\beta \cdot V_i}}{\sum_i e^{\beta \cdot V_i}}$$

To evaluate whether participants were expressing knowledge of the task's transition structure in their choice behavior, we compared this optimal model against simpler models. The simplest was a Q-learner, "Qfirst", with no knowledge of the transition structure. Because there were only on the order of 35 trials per session, it would be impossible to learn an action value representation over the entire space of 6 starting states × 16 actions. Meanwhile, it would be impossible to use the same set of Q-values to make predictions from future states without knowing the transition structure. "Qfirst" tackled these problems by only making predictions about the first move in each trial, reducing the number of Q-values to 6 × 2 = 12. Based on the final reward *M* obtained at the end of each trial, the Q-value for the chosen first action *c* was updated:

$$Q_{s,c} \leftarrow Q_{s,c} + \alpha \cdot \left(\gamma \cdot \left(M + \max_a Q_{s',a}\right) - Q_{s,c}\right)$$

where α was a learning rate, γ was a discount rate, *s* was the starting state for the trial, and the action *c* led to state *s*'. The first move on each trial was selected by softmax over the Q-values of the two possible actions:

$$P_i = \varepsilon + (1 - 2\varepsilon) \cdot \frac{e^{\beta \cdot Q_{s,i}}}{\sum_i e^{\beta \cdot Q_{s,i}}}$$

The remaining three moves in the trial were selected uniformly at random.

The third model, "Qall", relaxed the requirement that the agent have no knowledge of the transition structure. By relaxing this requirement we allowed the model to make predictions about all four moves in each trial. The best action (i.e., up or down) for move 2, 3 or 4 in a trial depended on what state the agent occupied based on its earlier moves in the trial. The model selected moves 2, 3 and 4 based on the participant's actually chosen, rather than the model's preferred, earlier moves, which allowed the model to make predictions about subsequent moves even if it mispredicted the early moves. Q-value updating and action selection worked the same as in Qfirst, except that Q-values for all four chosen moves were updated, and all four moves were selected (the total probability for a trial was the product of the probabilities for each of these moves).

The fourth model, "Greedy", implemented another strategy we considered *a priori* plausible. First, it tracked the reward values ρ_i for each of the six states, as in the Plan model. Second, it chose greedily at each move the state that led to the largest cumulative total *after that move*, taking negs into account:

$$V_{up} = -1^{neg_{up}} \cdot (CR + \rho_{up})$$

where neg_{up} was a binary variable indicating whether the state that would be reached by taking the up action was neg on this trial, CR was the cumulative reward gained on the trial before this move, and ρ_{up} was the reward value of the state that would be reached by taking the up action. Greedy therefore also used knowledge of the task's transition structure. Action selection was again by softmax, between V_{up} and V_{down}.

The parameters of all the models were fit using a genetic algorithm ('ga' in Matlab) to maximize the likelihood of the data. To guard against local minima, ga was run three times and the best run selected (usually all three runs were very similar).

Model comparison was performed using the Bayesian Information Criterion (BIC). For each participants and for each model, the log likelihood L was evaluated at the best fitting parameters. The BIC score for that participant for that model was

$$-2 \log L + n_p \log n_t$$

where n_p was the number of free parameters for that model, and n_t was the number of trials for that participant. BIC scores were summed over participants for group-level model comparison.

MEG acquisition and pre-processing

MEG was recorded continuously at 600 samples/second using a whole-head 275-channel axial gradiometer system (CTF Omega, VSM MedTech, Canada), while participants sat upright inside the scanner. Continuous head localization was recorded with three fiducial coils at the nasion, left pre-auricular, and right pre-auricular points. The task script sent synchronizing triggers (outportb in Cogent) which were written to the MEG data file. Timings were corrected for approximately one frame (1/60 s) of lag between triggers and refreshing of the projected image, measured using a photodiode outside the task. A projector displayed the task on a screen ~80 cm in front of the participant. Participants made responses on three buttons (called “up”, “down” and “advance”) of a button box using the fingers they found most comfortable.

Raw data in CTF .meg4 format were read into MATLAB arrays using the function spm_eeg_convert from SPM12 (Wellcome Trust Centre for Neuroimaging, University College London). All subsequent analysis was performed using built-in functions in MATLAB R2015a/b, without SPM. The data were first resampled from 600 Hz to 100 Hz to conserve processing time and improve signal to noise ratio. Thus, data samples used for analysis were spaced every 10 ms. All data were then high-pass filtered at 0.5 Hz using a first-order IIR filter to remove slow drift.

Two approaches were used to reject artifacts. First, we excluded all sensors that had more than 10000 samples of detected eyeblink across all sessions. This also minimized eye movement artifacts. 134 sensors were left included in the analysis. Second, we rejected any data that exceeded seven units of mean absolute difference, plus ten samples before and after. Finally, as mentioned above, two sessions were entirely excluded due to large artifacts that could not be easily rejected according to these methods. All rejection criteria were fixed before the start of sequence analysis.

All analyses were performed directly on the filtered, cleaned MEG signal, consisting of a length 134 vector of samples every 10 ms, in units of femtoTesla. No time-frequency decomposition, baseline correction, source reconstruction, or other pre-processing was used.

Multivariate MEG analysis

Logistic regression models were trained using MATLAB's function lassoglm, with the arguments 'binomial', ('Alpha', 1), and ('Lambda', Lp) where Lp was the lasso penalty whose value was determined through a cross-validation procedure. A trained model k consisted of a single vector $\boldsymbol{\beta}_k$ with length 135: slope coefficients for each of the 134 sensors together with an intercept coefficient. For both training and testing regression models, all input data were scaled such that the 95th percentile of the absolute value of each channel was equal to 1.

For model k, the training data labelled as "1" were the trials in which visual object k was presented. The training data labelled as "0" were trials in which any of the other five objects were presented, as well as a set of "null" data. The null data were the same for each of the six regression models, and consisted of the data immediately before the onset of each visual object. Thus, each model was trained on approximately 16 positive examples and 183 negative examples (and 100 of these ~183 were the same for all models). Models were validated on labelled data using leave-one-out cross validation.

To estimate the number of meaningfully independent components in the data, we performed principal components analysis on the data used to train the classifier (from 200 ms after stimulus onset). We found that the number of components needed to explain 95% of the variance across trials ranged from 6 to 17 between subjects.

Sequenceness Measure

Subtracting reverse from forward sequenceness was motivated by the existence of a strong autocorrelation in the underlying MEG signal at short time-lags (as time lag increased, autocorrelation became negative due to highpass filtering). Because classifier outputs were correlated with one another, each pair of classifier outputs also had a corresponding peak in cross-correlation near zero time lag. Therefore, both forward and reverse sequenceness alone were strongly positive at short time lags (Figure S3). Taking the difference between forward and reverse sequenceness minimized the main effect of autocorrelation while maximizing detectability of a systematic forward or reverse sequence effect. However, it was also possible to compare either forward or reverse transitions against probabilities that would be expected if representations jumped across a transition that did not exist in the task ('null transitions'; for example from S_1, jumping to S_4). Although reverse transitions were greater than null transitions (Figure S3), this difference did not reach significance due to high variability in the differences between cross-correlations of unmatched pairs of states.

For the participants who had two sessions, regression models were trained separately for each session to account for the possibility of different head positions, and sequenceness measures from the two sessions were averaged together before computing group-level statistics.
