# Supplementary material for: Fast Sequences of Non-spatial State Representations in Humans
Source: Neuron. 2016 Jul 6;91(1):194–204. doi: 10.1016/j.neuron.2016.05.028 (PMC4942698; doi:10.1016/j.neuron.2016.05.028)
Supplement: Document S2. Article plus Supplemental Information [file mmc2.pdf]

Article

Neuron

Fast Sequences of Non-spatial State Representations in Humans

Highlights

- Task requires navigating between objects whose neural representations can be decoded
- After task has been learned, spontaneous MEG activity encodes reverse paths
- These spontaneous sequences are up to four states long
- Sequences might be cognate with rodent hippocampal "replay"

Authors

Zeb Kurth-Nelson,
Marcos Economides,
Raymond J. Dolan, Peter Dayan

Correspondence

zebkurthnelson@gmail.com

In Brief

The rat hippocampus spontaneously imagines past and future locations in rapid sequences (sometimes called "replay"), which link multiple locations into coherent neural representations. Kurth-Nelson et al. find similar sequences in humans, in a non-spatial task, suggesting they are a ubiquitous mechanism.

Kurth-Nelson et al., 2016, Neuron *91*, 194–204
July 6, 2016 © 2016 The Authors. Published by Elsevier Inc.
http://dx.doi.org/10.1016/j.neuron.2016.05.028

Neuron
Article

Fast Sequences of Non-spatial State Representations in Humans

Zeb Kurth-Nelson,[1,2,*] Marcos Economides,[2] Raymond J. Dolan,[1,2] and Peter Dayan[3]
[1]Max Planck-UCL Centre for Computational Psychiatry and Ageing Research, University College London, London WC1B 5EH, UK
[2]Wellcome Trust Centre for Neuroimaging, University College London, London WC1N 3BG, UK
[3]Gatsby Computational Neuroscience Unit, University College London, London W1T 4JG, UK
*Correspondence: zebkurthnelson@gmail.com
http://dx.doi.org/10.1016/j.neuron.2016.05.028

SUMMARY

Fast internally generated sequences of neural representations are suggested to support learning and online planning. However, these sequences have only been studied in the context of spatial tasks and never in humans. Here, we recorded magnetoencephalography (MEG) while human subjects performed a novel non-spatial reasoning task. The task required selecting paths through a set of six visual objects. We trained pattern classifiers on the MEG activity elicited by direct presentation of the visual objects alone and tested these classifiers on activity recorded during periods when no object was presented. During these object-free periods, the brain spontaneously visited representations of approximately four objects in fast sequences lasting on the order of 120 ms. These sequences followed backward trajectories along the permissible paths in the task. Thus, spontaneous fast sequential representation of states can be measured non-invasively in humans, and these sequences may be a fundamental feature of neural computation across tasks.

INTRODUCTION

Most areas of the brain are engaged in encoding and representing current sensory inputs, contexts, and motor outputs. However, neural activity can also be decoupled from current input to encode representations of past or possible future states. Such decoupling is argued to underpin memory, imagination, and planning (Buckner and Carroll, 2007; Buzsáki and Moser, 2013; Carr et al., 2011; Jadhav et al., 2012; van der Meer et al., 2012; Pezzulo et al., 2014; Pfeiffer and Foster, 2013; Wikenheiser and Redish, 2015).

A remarkable, but now well-established, finding is that the decoupled activity of populations of neurons sometimes takes the form of internally generated sequences that encode trajectories through past or possible future states. This phenomenon has been most studied in rodent hippocampus, where place cells that normally encode an organism's current position in space also spontaneously play out sequences of other positions (Diba and Buzsáki, 2007; Foster and Wilson, 2006; Louie and Wilson, 2001; Skaggs and McNaughton, 1996). Internally generated hippocampal sequences occur in two distinct physiological contexts, embedded within sharp-wave ripple events (e.g., Diba and Buzsáki, 2007) or nested within theta rhythm (e.g., Johnson and Redish, 2007). The relationship between sequences in these two contexts remains unknown (Schmidt and Redish, 2013), and here we discuss observations in both. Spontaneous hippocampal sequences have been observed in sleep and wakefulness and appear in a variety of spatial tasks (Davidson et al., 2009; Gupta et al., 2010; Karlsson and Frank, 2009; Lee and Wilson, 2002). Although less extensive, there is also evidence for fast spontaneous sequences outside of hippocampus (Euston et al., 2007; Hoffman and McNaughton, 2002). The interaction of cortical with hippocampal sequences is not yet understood, although in simultaneous recordings the hippocampus plays out what appears to be the same experience as visual cortex (Ji and Wilson, 2007).

Two, not mutually exclusive, classes of function are suggested for fast spontaneous sequences. First, in the context of learning, they may be part of a mechanism for consolidating or maintaining knowledge, particularly in cortex (Káli and Dayan, 2004; Louie and Wilson, 2001; Mnih et al., 2015; Siapas and Wilson, 1998). Temporal compression of sequences, relative to real experience, might bring distal events within a time frame within which synaptic plasticity mechanisms can operate, particularly those used for credit assignment (Foster and Wilson, 2006; Jensen and Lisman, 2005; Skaggs et al., 1996). Second, sequences may play a role in planning or look-ahead in decision making, either online or offline (Sutton, 1991). Sequences beginning at the animal's current location sometimes predict the path the animal will run in the immediate future (Pfeiffer and Foster, 2013; Wikenheiser and Redish, 2015). Concurrently, value signals emerge that are suggested to be a downstream consequence of such prospection (Lansink et al., 2009; van der Meer and Redish, 2009; van der Meer et al., 2010).

Despite the importance of fast spontaneous sequences, and their potential generality as a mechanism for learning and decision making (Buzsáki and Moser, 2013), they have so far only been studied in spatial tasks, and overwhelmingly in rodents. Our goal was to investigate spontaneous neural sequences in a non-spatial context in healthy human volunteers. Previously, we used multivariate analysis of magnetoencephalography (MEG) data to decode time-resolved representations of visual objects that were not currently being experienced (Kurth-Nelson

 © 2016 The Authors. Published by Elsevier Inc.
This is an open access article under the CC BY license (http://creativecommons.org/licenses/by/4.0/).

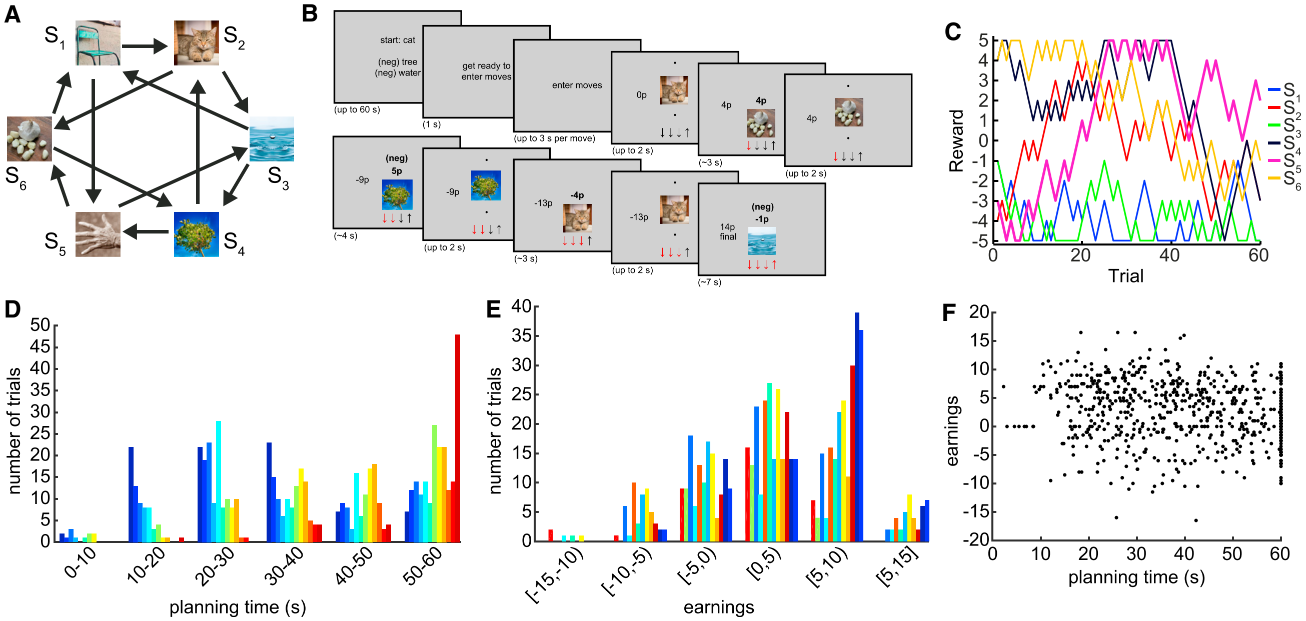

Figure 1. Task Design

Participants navigated between six states (S_1–S_6), each corresponding to a visual object.

(A) The states were linked to one another as shown, although the visual objects assigned to each state number were randomized across participants.

(B) On each trial, participants began in a random state and were permitted four moves. They had up to 60 s to plan these four moves. The four moves were then entered rapidly with no feedback. After rapidly entering their chosen sequence of moves, participants were required to play out this sequence. While playing out the sequence, the objects and their associated reward were visible.

(C) The reward associated with each state drifted slowly over trials. The total reward earned in each trial was cumulative of the reward collected along the path. When a “neg” state was reached, it caused the sign of the cumulative collected reward to flip (negative to positive and vice versa).

(D) Distributions over participants of time used to plan, up to a maximum permitted 60 s. Each color corresponds to a unique participant, sorted by mean planning time.

(E) Distributions over participants of money earned per trial, relative to the expected earnings of a random policy on that trial. Each color corresponds to the same participant as in (D).

(F) Trials with greater earnings tended to have shorter planning time ($p = 0.002$ by linear mixed model). However, participants with shorter mean planning time did not have higher mean earnings. Each point is a trial, with all participants shown together.

et al., 2015). We therefore reasoned that it might be possible to detect spontaneous fast sequences using MEG in a non-spatial task in which states were defined by decodeable visual objects.

RESULTS

Task

Participants performed a novel six-state non-spatial navigation task. Each state was defined by a unique visual object and associated with a varying amount of reward, ranging from −5 to +5 pence. From each state, two choices were available (called “up” and “down”), each of which led to a different state (Figures 1A and 1B). Before scanning, participants were trained to criterion on the structure of the task. On each trial during scanning, participants started from a random state and were asked to enter a sequence of four moves with the goal of collecting as much reward as possible. This sequence of moves defined a path around the maze. To discourage simple stimulus-response learning strategies, the task had two additional features. First, reward amounts changed by −1p, 0p, or 1p at random per trial (Figure 1C). Second, at the beginning of each trial, participants were informed that two (randomly selected) states would be “neg,” meaning that reaching either of these states would multiply the trial’s cumulative reward by −1. Importantly, participants never saw a bird’s-eye view of the maze and only experienced one visual object at a time. At debriefing, no participant reported conceiving the relationships between objects in a spatial manner.

We reasoned that neural sequences following the transition structure of the task could only occur if participants in fact learned the transition structure. Although the training and task were designed to encourage such learning, we sought to confirm that it did actually occur. First, in 2–3 days of training before scanning, all participants were required to reach a criterion of 100% accuracy on a set of automated quiz questions that probed knowledge of the transition structure (e.g., “if you start at horn and go up, where will you be?”). Second, in debriefing post scanning, all participants reported a subjective experience of deploying knowledge of transitions for planning. For example, “I didn’t always manage to think four steps ahead, but sometimes I did” and “I tried to make a four-step plan, but sometimes missed the negative on the fourth step.” Third, model comparison on behavioral choice data strongly favored models that planned with the task’s structure over stimulus-response models (Figure S1).

After the starting state was revealed at the beginning of a trial, participants were allowed up to 60 s for planning, with the possibility of entering moves earlier by pressing a button. The distribution of actual planning times is shown in Figure 1D, with a median of 38.9 s (interquartile range [IQR] = 27.3 s). For more details of the timings within each trial, see Experimental Procedures.

After the planning period, participants had to pre-enter their four moves quickly or face a monetary penalty. During this pre-entry, the sole feedback was the appearance of an "up" or "down" arrow, displaying each move that they selected. The mean reaction time to pre-enter the first move in the slowest participant was 760 ± 50 ms and in the fastest participant was 304 ± 21 ms. After pre-entry, participants were required to execute the same sequence of moves they had just pre-entered. During execution, the visual objects associated with each state were displayed, and as they executed each move, the visual object representing the current state faded into the object representing the next state. The mean reaction time to execute the first move in the slowest participant was 998 ± 63 ms and in the fastest participant was 408 ± 18 ms. As each visual object appeared during execution, its current reward value (from −5 to +5 pence) appeared alongside it. The cumulative total trial earnings were displayed continuously during the execution phase and were updated as each visual object appeared.

Task performance, in terms of money earned, was significantly higher than chance for 9/12 participants (Figure 1E). Between trials, there was a significant relationship between planning time and earnings, with more money earned on trials with shorter planning time (Figure 1F; $p = 0.002$ by mixed effects linear regression).

Multivariate Models of State Representations

For each subject and for each visual object independently, we trained a lasso-regularized logistic regression model to recognize instantaneous spatial patterns of MEG elicited by direct visual presentations of the object. The lasso penalty encouraged sparsity and tended to select occipital and posterior temporal sensors as useful features (Figure S2). Data used to train the regression models were taken from a secondary task in which the objects were presented multiple times in random order (see Experimental Procedures for details). Based on findings from our previous work (Kurth-Nelson et al., 2015), all models were trained only on MEG data recorded 200 ms after visual object onset.

Models were cross-validated on training data to confirm that they captured essential object-related features in the MEG signal. When models trained to recognize object *k* were tested on left-out data whose true class was *k*, the predicted probability reached 0.19 ± 0.02, peaking at 200 ms post stimulus onset. When models trained to recognize object *k* were tested on left-out data whose true class was not *k*, the predicted probability reached 0.08 ± 0.003 (Figure 2A). To ascertain that the models correctly decoded the objects, we also performed the same analysis 100 times with randomly shuffled state labels. In each shuffle, we used as a maximal statistic the maximum predicted probability over states and over time, thereby conservatively controlling for multiple comparisons. This yielded a set of 100 maximal statistics, and we used the 95th percentile of that set as a $p = 0.05$ significance threshold (dashed line in Figure 2A). All models' actual predicted probabilities exceeded this threshold when the true stimulus was the same as what they had been trained to detect.

To test the specificity of the models, we analyzed prediction accuracies. The set of models together could be treated as making a categorical prediction about the class of the left-out data, by identifying the model with highest output probability on left-out data. The cross-validated prediction accuracy reached up to 53.7% ± 3.8%, where chance was 16.7% (Figure 2B). We also performed a similar shuffling procedure as above, using the maximum accuracy over time as the maximal statistic, and found that the true classification accuracy exceeded 100/100 shuffles.

Sequences in Decoded Object Representations

We applied the trained regression models to MEG data collected in the planning phase of each trial. As already described, this planning phase was a period that varied in duration from 2 to 60 s (cf. Figure 1D for distribution of times) during which no visual object was onscreen. Each 10 ms time bin of these data was independently input to each of the six regression models, yielding six time series of probabilities for each trial (example trial shown in Figure 3A). The probability in time bin *t* from model *k* quantified the degree to which the spatial pattern of MEG activity at time *t* resembled the evoked neural response to visual object *k*.

We next asked whether these time series contained sequences that followed possible paths in the behavioral task based upon a measure we refer to as "sequenceness." For example, in the task there was a potential transition from state S_1 to state S_2. Sequenceness quantified whether a decoded neural representation of state S_1 was likely to be followed by a decoded representation of state S_2 or, in case of reverse sequences, whether S_2 would be followed by S_1. We operationalized sequenceness using a cross-correlation measure (see Experimental Procedures for details).

Sequenceness could either be forward (e.g., a representation of state S_2 followed that of state S_1) or reverse (e.g., S_2 preceded S_1). We observed a peak in reverse sequenceness at 40 ms of lag (Figure 3B). This signifies that if a neural representation of state *k* was active at time *t*, then a representation of one or both of the states with transitions to *k* was active around time *t* + 40 ms. We tested whether sequenceness at 40 ms lag was significantly different than zero using a multilevel model with a single fixed intercept term and one random intercept term for each participant. The fixed intercept term was estimated at -8.3×10^{-3} ($p = 0.0015$, two tailed). Next, in order to avoid relying on the assumptions of this model, we used a nonparametric method, shuffling the state identities 28 times to generate a null distribution of sequenceness (see Experimental Procedures for details). By taking the peak of the absolute value of each shuffle across all possible lags, and then taking the maximum of these peaks across shuffles, we obtained a conservative two-tailed significance threshold at approximately $p = 1/28 \approx 0.036$. The real data exceeded this threshold from 20 to 70 ms of lag. We note that although the relatively small sample size in our study

warrants caution, the consistency of sequenceness between participants (Figure 3C) was both striking and reassuring.

The logistic regression models had a free parameter defining the amount of regularization. To ensure the results were not an artifact of selecting a particular parameter value, the data shown in Figure 3B, and used for both parametric and non-parametric statistics described above, were obtained by cross-validating over this parameter. The parameter used to calculate sequenceness for each subject was the value that gave the strongest average sequenceness in the other 11 participants. The best value of the parameter was relatively consistent between cross-validation folds (Figure 3B, inset). Finally, the reverse sequenceness effect was stable across trials within each session (Figure 3D). Examples of individual sequence events, each lasting on the order of 100–200 ms, are shown in Figure 4.

Length of Sequences

We next asked whether the reverse sequenceness effect was driven by pairs of object representations appearing in isolated sequences (i.e., length 2 sequences) or by longer contiguous sequences. We explored sequences of length 3, 4, and 5. A length 3 reverse sequence would occur if, for example, state S_6 had high probability at time t, state S_5 had high probability at time t + 40 ms, and state S_4 had high probability at time t + 80 ms. The length *n* sequenceness measure we used was a generalization of the cross-correlation measure used to detect length 2 sequences (see Supplemental Experimental Procedures for details). Because this method does not allow direct comparison of effect magnitude between different sequence lengths, we compared effect reliability using the same multilevel model described above. The fixed effect intercept term, which quantifies overall sequenceness, was reliably different from zero at 40 ms state-to-state lag for length 3 ($p = 0.009$) and length 4 ($p = 0.004$) sequences. The intercept term was not different from zero for length 5 sequences ($p = 0.4$). We conclude that spontaneous state representations tended to occur as fast sequences of up to four consecutive states (Figure 5).

Given that the sequence effect was strongest at a state-to-state lag of 40 ms, we estimated that an entire 4-state sequence, consisting of three transitions, lasted on the order of 120 ms. As in most rodent studies (e.g., Diba and Buzsáki, 2007; Dragoi and Tonegawa, 2011; Gupta et al., 2010; Ji and Wilson, 2007), these sequences were compressed in time relative to real experience. The most visually striking marker of state change during move execution was the visual cross-fade between objects, which took 350 ms. Relative to this, sequences were temporally compressed by a factor of 9. Meanwhile, the duration of an entire

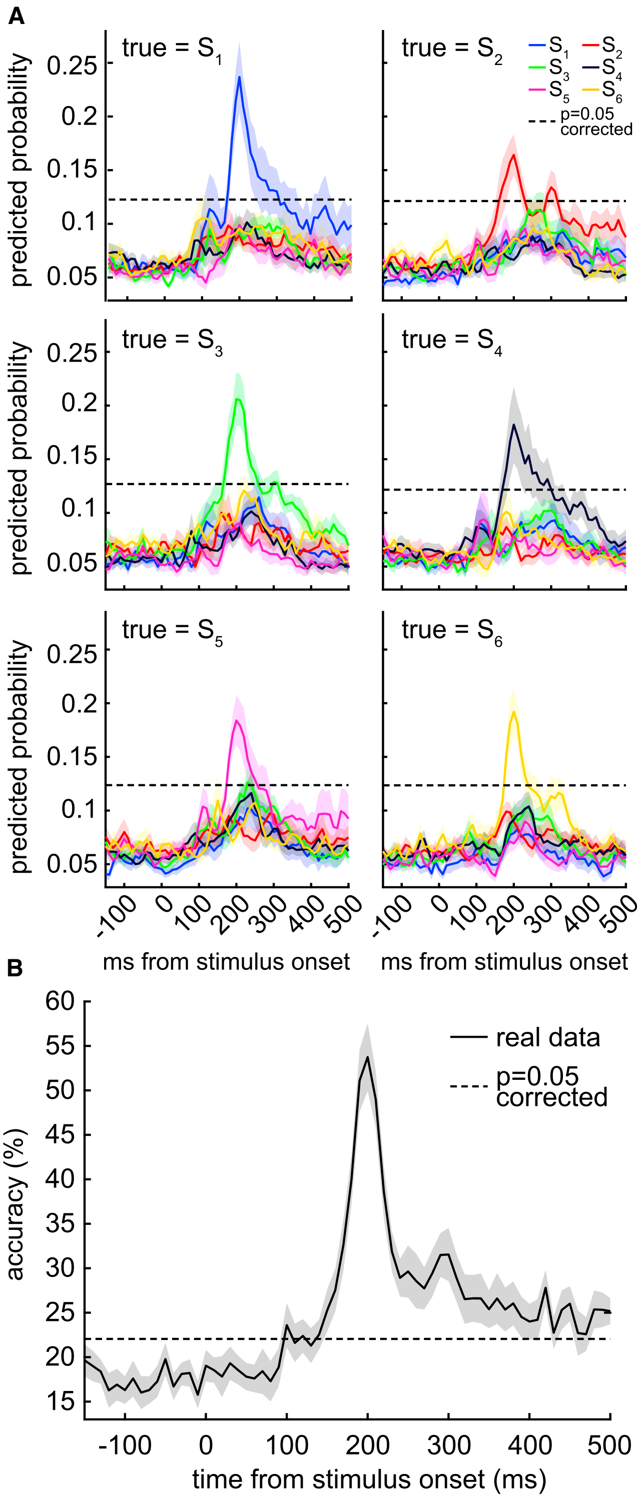

Figure 2. Lasso Logistic Regression Models Trained on Direct Presentations of Visual Objects

For each participant, a separate regression model was trained to recognize each visual object.

(A) We used leave-one-out cross-validation to test the generalizability of the learned models. These plots show the average probabilities output by the six models in cross-validation, with each panel corresponding to a different true state and each colored line showing the output of a different model. Because the models were trained only at 200 ms, correct prediction had a briefer peak than in other studies where classifiers were both trained and tested at every time point (e.g., Kurth-Nelson et al., 2015).

(B) Prediction accuracy could be estimated by treating the index of the model with highest probability output as the predicted object. In cross-validation, the prediction accuracy reached 53.7% ± 3.8%, where chance was 16.7%. Dashed lines show 95% of empirical null distribution obtained by shuffling state labels. Shading indicates SEM.

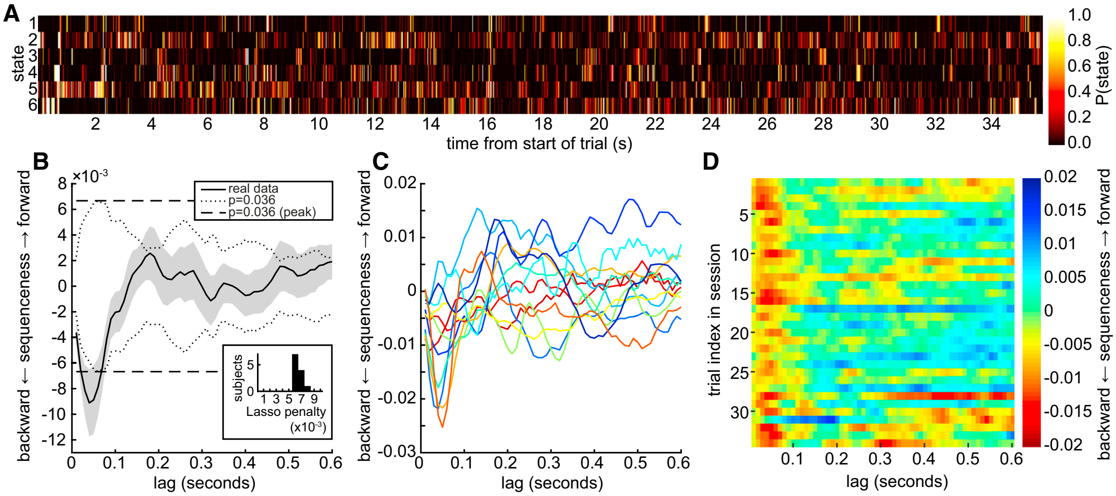

Figure 3. Decoded State Representations follow Reverse Sequences through the Maze

(A) Probability time series output from the six regression models on a single example trial (one of 603 trials analyzed). Time zero corresponds to the start of the planning phase of the trial. We were interested in whether these time series contained any sequences following the transition structure of the task.

(B) The y axis is the degree to which a neural representation of a state tended to be followed by (positive *y*) or preceded by (negative *y*) a neural representation of a successive state in the task (cf. Figure 1A), a measure we call sequenceness. This was quantified independently at all possible state-to-state lags (x axis). There was strong evidence for reverse sequenceness at around 40 ms of state-to-state lag. This effect was significant by parametric mixed intercept model (p = 0.0015 at 40 ms, not corrected for multiple lags) and by a non-parametric test based on shuffling the state identities. Dotted line shows non-parametric p = 0.036 threshold independently at each lag. Dashed line shows the peak of shuffles over all lags, which corrects for multiple lags, and was exceeded by the real data from 20 to 70 ms of lag. Inset: the lasso penalty for logistic regression models was selected by leave-one-out cross-validation over subjects to prevent over-fitting. Histogram shows distribution of lasso penalties across subjects.

(C) 12/12 participants had reverse sequenceness at some time between 20 and 70 ms. Each line is an individual participant's sequenceness plotted for all lags. Each participant is shown in the same color as in Figure 1. Individual data are shown at the group mode lasso penalty, 0.006.

(D) The sequence effect was stable over trials within session (averaged over participants). Trials beyond 34 are not shown because few sessions exceeded 34 trials.

state transition, including the time to display reward information, varied from approximately 1 to 4 s. Relative to this benchmark, sequences were temporally compressed by a factor of ~25–100.

Negative Results

Between participants, there was no significant relationship between planning time and earnings (p = 0.3 by regression on subject means), between planning time and sequenceness (p = 0.5 by regression on subject means), or between earnings and sequenceness (p = 0.2 by regression on subject means). We note that the absence of evidence in inter-individual differences should be interpreted cautiously, as the small sample size of the study was ill-suited to detect such differences.

Finally, we asked whether we could detect any trial-by-trial relationship between neural sequences and behavior. Across trials, we regressed the magnitude of sequenceness against the actual earnings and found no effect (p = 0.83 by linear mixed effects). We also regressed the magnitude of sequenceness against planning time on the same trial and found no effect (p = 0.27 by linear mixed effects).

As a more specific test, we asked whether the specific sequences encoded in the MEG data on a trial were predictive of the moves a subject would actually make on that trial. To test this, we ran a similar analysis to the main sequence analysis, but looking at the cross-correlation between individual pairs of states at 40 ms lag. For each state pair, we again subtracted reverse ($S_j \rightarrow S_i$) from forward ($S_i \rightarrow S_j$) cross-correlations. This yielded a magnitude of "sequenceness" for each individual pair (Figure S5). At the group level, there was no evidence for differences between pairs (one-way ANOVA, F(11,132) = 0.92, p = 0.52), as would be expected because the mapping from state to visual object was randomized between participants. We then tested whether trial-by-trial variability in these individual pairs related to behavior. On each trial, participants chose a sequence of four moves, or four $i \rightarrow j$ tuples. We tested whether the magnitude of sequenceness for individual tuples in the MEG data was greater for chosen tuples than unchosen tuples and found no effect (p = 0.85 by mixed intercept model). We also repeated the same analysis but restricted to the first chosen move in each trial; again, there was no difference between neural sequenceness of chosen tuples versus unchosen tuples (p = 0.15). Finally, we evaluated whether the magnitude of sequenceness for individual tuples in the MEG data was greater for chosen tuples than unchosen tuples on the previous trial and again found no effect when analyzing all four moves (p = 0.62) or only the first chosen move (p = 0.42).

DISCUSSION

We show that spontaneous MEG activity plays out fast sequences of state representations, after participants have learned a non-spatial navigation task based on one-way connections

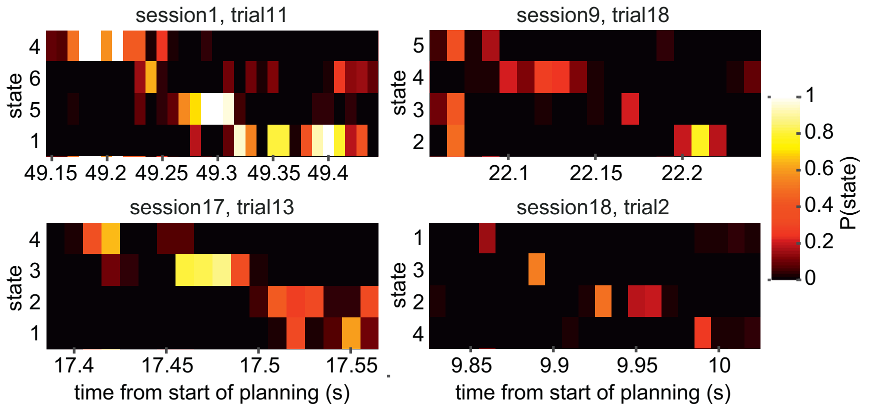

Figure 4. Example Sequence Events

Time progresses rightward along the x axis. Each row depicts the probability outputs of one regression model, and the rows are sorted top to bottom to follow the reverse order of transitions in the task. Each example is from a different participant. We manually selected examples on the basis of looking good, so they should not be taken as statistically meaningful.

between these states. These sequences formed trajectories of up to four states that progressed backward through the connections of the task. The sequences had a state-to-state lag of 40 ms, meaning that a whole trajectory lasted on the order of 120 ms. Although spontaneous sequences have been reliably observed in rodent spatial navigation experiments, this is the first report, to our knowledge, of such sequences in humans, as well as the first in a non-spatial task setting (although sequences in spatial tasks can be modulated by information about non-spatial context; Takahashi, 2015). These results suggest that fast non-local sequences may be a fundamental neural mechanism in decision making that is conserved across species and across problem domains.

Non-spatial Sequences and Hippocampus

Although all previous observations of fast spontaneous sequences have used spatial tasks, most of the functions suggested for these sequences in learning and decision making apply to non-spatial as well as spatial settings. Accordingly, a role for fast neural state sequences has been hypothesized in a range of cognitive processes (Buzsáki and Moser, 2013; Carr et al., 2011; Pezzulo et al., 2014).

Tolman made the seminal suggestion that an agent should build abstract cognitive maps for non-spatial as well as spatial tasks (Tolman, 1948). A computational view has emerged that this is a role played by the hippocampus and associated brain structures in a wide range of cognitive problems (Allen et al., 2016; Collin et al., 2015; Eichenbaum and Cohen, 2014; Killian et al., 2012; Milivojevic and Doeller, 2013; Muller et al., 1996; O'Keefe and Nadel, 1978; Redish, 1999; Schapiro et al., 2012; Shapiro and Eichenbaum, 1999; Takahashi, 2013; Tavares et al., 2015). If so, the hippocampus should express sequences in non-spatial tasks as it does in spatial tasks.

However, although MEG contains signals of hippocampal origin (Dalal et al., 2013), and several studies have reported source localizing MEG activity to hippocampus (e.g., Cornwell et al., 2008; Guitart-Masip et al., 2013), these signals are relatively difficult to detect, rendering it unlikely that the sequences we recorded arose directly from hippocampus. First, a fall-off of magnetic field strength with the square of distance from neural sources ensures cortical activity dominates the MEG signal (Hämäläinen et al., 1993; Riggs et al., 2009; Stephen et al., 2005). Second, our classifiers were trained on evoked visual responses shortly after visual stimulus onset, which can be assumed to reflect most strongly activity arising in a cortical visual processing stream. Third, the sensors used by the regularized regression models were mostly occipital and posterior temporal sensors in a pattern different from that reported for putative hippocampal activity (Dalal et al., 2013; Guitart-Masip et al., 2013).

The fact that our classifiers were trained on visual evoked activity makes it most likely that the observed sequences corresponded to reactivation of visual representations. In humans, the pattern of cortical activity observed during direct experience with a sensory object is at least partially reinstated when the object is retrieved or remembered (Danker and Anderson, 2010; Kuhl and Chun, 2014; Nyberg et al., 2000; Polyn et al., 2005; Wimmer and Shohamy, 2012). This reactivation is implicated in model-based reasoning, and we have previously shown that it can be tracked at fast timescales with MEG (Kurth-Nelson et al., 2015).

Since hippocampus drives cortical representations during retrieval (e.g., Tanaka et al., 2014), it is possible that sequences generated in hippocampus (perhaps during sharp-wave ripples [SWRs]) might drive sequences of cortical activity detected by our classifiers. This would be consistent with a coupling observed between hippocampus and cortex during SWRs (Siapas and Wilson, 1998; Sirota et al., 2003; Wierzynski et al., 2009) and the coordination of hippocampal and cortical sequences (Ji and Wilson, 2007). However, this remains speculative, and our data provide no direct evidence of an upstream role for hippocampus. It is entirely possible that the sequences we observed originated from intrinsic cortical dynamics, in keeping with prior observations that spontaneous space-related sequences occur in a variety of cortical areas in rats and primates (Euston et al., 2007; Hoffman and McNaughton, 2002; Ji and Wilson, 2007).

Forward versus Reverse Sequences

Our main analysis was based on subtracting reverse from forward sequenceness. At most latencies, there was no difference between the two. However, at a lag of around 40 ms, there was much stronger expression of reverse compared to forward sequenceness. In principle, this effect at 40 ms could reflect either an increase in reverse sequences or a reduction in forward sequences, relative to baseline. However, we think the latter unlikely because it would imply a consistent positive amount of forward sequenceness at all other latencies.

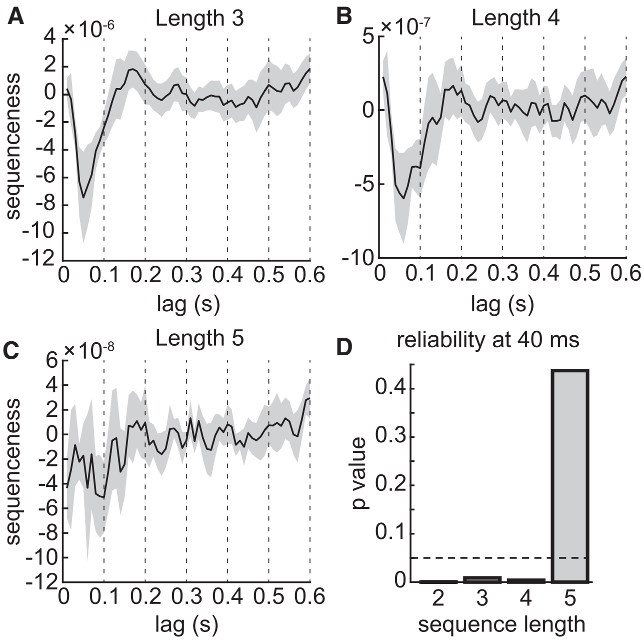

Figure 5. Sequences Are Reliable up to Length 4, but Not Length 5

(A–C) The sequenceness measure shown in Figure 3B could be generalized to longer sequences. These plots show the degree to which decoded state representations appeared in consecutive sequences of length 3 (A), 4 (B), or 5 (C).

(D) The un-normalized inner products used to detect higher-order sequences did not bear direct comparison between different sequence length, but could be compared through their reliabilities, expressed as p values of the mixed intercept model described in the main text. Length 3 and length 4 sequences were nearly as reliable as the length 2 sequences shown in Figure 3, but no length 5 sequences were detectable at 40 ms lag. (A trend toward length 5 reverse sequenceness appeared at a slower state-to-state lag of 100 ms but did not reach significance.) A dashed line is drawn at $p = 0.05$ for reference.

Sequences within hippocampal SWRs in rats, which occur when the animal is pausing or resting, have been observed in both forward and reverse order (Diba and Buzsáki, 2007; Foster and Wilson, 2006; Gupta et al., 2010; Nádasdy et al., 1999; Skaggs and McNaughton, 1996). Theta sequences, which occur during active behavior, are nearly always forward (Foster and Wilson, 2007; Gupta et al., 2012; Johnson and Redish, 2007; Wikenheiser and Redish, 2013, 2015). This raises the question of the behavioral state of our subjects during the planning period of up to 60 s when sequences were observed. Given the relatively long time permitted for planning (up to 60 s) in our task, and the high attentional demands at other times within the task, it is entirely plausible that sequence events seen in our study corresponded to moments when people were pausing from actively calculating moves.

Another interesting question concerns whether neural sequences are "local" or "remote," meaning whether or not they represent a trajectory that includes the current state of the agent. In rodents, theta sequences contain (but do not necessarily begin or end at) the current location of the animal (Gupta et al., 2012; Johnson and Redish, 2007; Wikenheiser and Redish, 2015). By contrast, SWR sequences sometimes include the current location of the animal (Diba and Buzsáki, 2007; Foster and Wilson, 2006) but also encode trajectories that do not include the current location of the animal (Davidson et al., 2009; Gupta et al., 2010; Karlsson and Frank, 2009). In our MEG data, sequences containing the state pair of the participant's first move on the trial were no more common than sequences not containing this pair, suggesting that sequences were as likely to be initiated remotely as locally.

Length of Sequences

Hippocampal sequence events in rodents last on the order of 50–200 ms, whether they occur in theta (Wikenheiser and Redish, 2015) or in SWRs (Diba and Buzsáki, 2007). This is similar to the 120 ms length we estimate for a four-state sequence event in our data. In rodent navigation experiments, states are defined continuously in space rather than discretely as in our maze, which does not admit a direct comparison with the number of states visited.

Retrospection versus Prospection

In the rodent literature, a number of functions are imputed in common to both forward and reverse sequences. This is particularly true of retrospective functions including assigning credit to recent experience (Foster and Wilson, 2006), consolidating memory (Carr et al., 2011), or active memory maintenance (Káli and Dayan, 2004). Consistent with a role in learning and memory, new experiences boost the frequency of SWRs (Eschenko et al., 2008) and boost coordination of neuronal activity within these SWRs (Cheng and Frank, 2008), while disruption of SWRs harms memory performance (Girardeau et al., 2009; Jadhav et al., 2012). The complexity of our task raises the possibility that consolidating knowledge of task structure was ongoing during performance, and the sequences we observed in MEG might play a role in this process.

Sequences ascribed prospective functions have most often been of a forward variety (Johnson and Redish, 2007; van der Meer and Redish, 2009; van der Meer et al., 2010; Pfeiffer and Foster, 2013; Wikenheiser and Redish, 2015). The reverse order of our sequences, along with the fact that they were not more likely than chance to contain the subsequently chosen state sequence, might argue against a prospective function. However, reverse sequences with a possibly prospective function were observed in Ólafsdóttir et al. (2015), and some rodent studies postulating a prospective function for sequences observed no simple relationship between neural sequences and upcoming behavior (e.g., Johnson and Redish, 2007). It is perfectly possible to plan backward instead of forward (e.g., LaValle, 2006), and it would be premature to conclude that the sequences we observed have purely retrospective functions. One interesting future test would be to collect MEG data during times when participants do not have access to information they need to plan an upcoming action. If sequences are in fact used for online planning, we would expect they should not occur when participants are not actively engaged in planning.

Intermediate between retrospective and prospective accounts, the observed sequences might also play a role in working memory. Even after training, participants reported that recalling the transitions was effortful. Fast neural sequences

could reflect a form of online working memory, interfacing between long-term memory and planning mechanisms. Another possible intermediate function is offline planning (Shohamy and Daw, 2015; Sutton, 1991), where values of paths or sub-paths might be calculated and stored in anticipation of future use.

Conclusions

Our results highlight the power of multivariate analysis of MEG data to trace the trajectories of fast-evolving neural representations in humans. In addition to being non-invasive, multivariate MEG has an added benefit of tracking representations that may be distributed across wide cortical areas. Applying this methodology has enabled us to provide the first evidence for fast spontaneous sequences of state representations in the human brain. Additionally the findings demonstrate this neural motif outside the spatial domain. Thus, fast sequences appear to be a fundamental principle of neural computation in a range of cognitive domains.

EXPERIMENTAL PROCEDURES

Participants

12 adults aged 18–31 participated in the experiment, recruited from the UCL Institute of Cognitive Neuroscience subject pool and from a mailing list for MSc students. Six were female and two were left-handed. All participants had normal or corrected-to-normal vision and had no history of psychiatric or neurological disorders. Eight of the 12 participants underwent two scanning sessions, for a total of 20 recorded sessions. Two of these sessions were excluded before the start of analysis owing to large artifacts, leaving 18 analyzed sessions. All participants provided written informed consent and consent to publish prior to start of the experiment, which was approved by the Research Ethics Committee at University College London (UK), under ethics number 1825/005.

Task

In the MEG scanner, participants performed a 6-state sequential reasoning task inspired by Huys et al. (2012, 2015) but designed with the additional criterion of encouraging mental representation of the visual objects that identified each state. The task was implemented in MATLAB (MathWorks) using Cogent (Wellcome Trust Centre for Neuroimaging, University College London). Each trial began with the participant being placed at a randomly selected state within the maze. From this state, they were permitted four sequential moves with the instructed aim of maximizing their earnings. From each state, a move constituted one of two possible choices, called "up" and "down" (so-called for simplicity of button pressing, although there was no meaningful spatial relationship between the states). Each of these choices deterministically led to a different next state. Only one state was ever viewed at a time, and participants never saw a bird's-eye view of the maze.

Each state provided a monetary outcome of between −5 and +5 pence. The reward for each state drifted independently at random by −1, 0, or +1 pence on each trial. Upon reaching a state, the state's current reward value was added to the participant's running total for that trial. This running total was also displayed on the screen while moves were being executed. Finally, in each trial, two randomly selected states were designated as "neg" states. When a neg state was reached, first its reward value was added to the running total for the trial as usual, but then the sign of the running total for the trial was flipped (e.g., −9 became +9 and vice versa). The identities of the neg states were signaled in text at the beginning of each trial. In many trials, the optimal strategy involved the use of one or two neg states. Two neg states could be used within a trial to reach a positive total reward, or a single neg state could be used in conjunction with negative state reward.

On each trial, participants were first shown in text the names of the starting state and the two neg states and allowed up to 60 s to plan. After the end of the planning period, participants were faced with a blank screen upon which they could pre-enter their chosen sequence of four moves. They were allowed up to 3 s to enter the first move and 1 s for each of the last three moves. As they pre-entered each move, a corresponding up or down arrow appeared on the screen for confirmation, but no visual objects were shown. After pre-entering all four moves, the visual object corresponding to the starting state of this trial appeared. Participants were then required to repeat the sequence of moves they had pre-entered. As they executed each move, the visual object shown on the screen changed to reflect the corresponding state transition. Up to 10 s was permitted to execute each move. Executing each move was followed by 350 ms of animated cross-fade transition between visual objects, followed by 500 ms pause, followed by the current reward amount of the new state displayed for 1,000 ms, followed by the total trial earnings being updated and displayed for 1,000 ms, followed by a neg and corresponding change to total trial earnings, if any, being displayed for 1,000 ms. If this was the final move of the trial, the final reward for the trial was then displayed for 3,000 ms; otherwise, the next move could be entered.

The task design was motivated by a wish to encourage participants to learn and use the transition structure of the task, instead of relying on simple choice strategies like repeating reinforced actions. We reasoned that engaging participants with the transition structure would afford us the best chance of detecting neural sequences reflecting this structure. Two other features of the task design were also intended to meet this purpose. First, trials were generated such that simple choice strategies would yield much lower payouts than optimal planning strategies (see Supplemental Experimental Procedures for more detail). Second, pre-entry of the four sequential moves on each trial was made in the absence of feedback about the consequences of those moves until all four had been entered. This meant that participants had to anticipate where move m would lead in order to make a good decision on move $m + 1$.

Each of the six states in the maze was a unique visual object. For each participant, the six objects were drawn randomly from a set of ten objects (bird, bread, cat, chair, garlic, hammer, hand, horn, tree, water), and the six chosen objects were randomly assigned to the six states of the maze.

After the 6-state reasoning task, participants completed a secondary task while still in the scanner. This task was designed to elicit neural representations of known stimuli, which could be used to train classification models. In this secondary task, the name of a visual object appeared in text for a variable duration of 1,500 to 3,000 ms, followed immediately by the visual object itself. On 20% of trials, the object was upside-down. To maintain attention, participants were instructed to press one button if the object was correct-side-up, and a different button if it was upside-down. Once the participant pressed a button, the object was replaced with a green fixation cross if the response was correct and a red cross if the response was incorrect. This was followed by a variable length intertrial interval of 700 to 1,700 ms. Each session included 125 trials of the secondary task, with approximately 16 correct side-up presentations of each visual object. Only correct-side-up presentations were used for classifier training. The trial order was randomized for each participant. Per participant, the visual objects used were the same six objects used in the main task.

Behavior Analysis

We fit four models to explain participants' choices. "Plan" used a full-depth tree search to calculate the value of each of the 16 possible sequences of four moves on each trial. This model represented optimal behavior on the task. "Qfirst" used no knowledge of the task's structure but learned Q-values for the 12 available state-action pairs based only on the first move in each trial. "Qall" also learned Q-values for the 12 available state-action pairs but exploited knowledge of the task structure to evaluate actions and update Q-values for all four moves in each trial. "Greedy" also used knowledge of the task structure but, rather than learning Q-values, selected each move in sequence by maximizing expected return locally from that move. Details of models and model comparison are given in the Supplemental Experimental Procedures.

MEG Acquisition and Pre-processing

MEG was recorded continuously at 600 samples/second using a whole-head 275-channel axial gradiometer system (CTF Omega, VSM MedTech), while

participants sat upright inside the scanner. Participants made responses on three buttons (called "up," "down," and "advance") of a button box using the fingers they found most comfortable.

The data were resampled from 600 Hz to 100 Hz to conserve processing time and improve signal to noise ratio. Thus, data samples used for analysis were spaced every 10 ms. All data were then high-pass filtered at 0.5 Hz using a first-order IIR filter to remove slow drift. All analyses were performed directly on the filtered, cleaned MEG signal, consisting of a length 134 vector of samples every 10 ms, in units of femtotesla.

Multivariate MEG Analysis

Lasso-regularized logistic regression models were trained on MEG data elicited by direct presentations of the visual objects. These presentations were taken from the secondary task that succeeded the 6-state reasoning task in the scanner, specifically the data 200 ms following stimulus onset. This 200 ms time point was selected based on observations from our previous work (Kurth-Nelson et al., 2015), which showed that when object representations are retrieved, the reinstated spatial pattern used in value reasoning is most similar to the pattern observed 200 ms after onset of direct object presentation. This constitutes the only available information to our knowledge about which of the time series of patterns evoked by direct object experience might be reinstated during decision making.

Models were verified on training data through cross-validation. In each cross-validation fold, we partitioned the data randomly, under constraints that ensured balanced classes for training: (1) each object had at least one left-out trial, (2) each object had the same number of left-in trials, and (3) the number of left-out trials was minimized subject to the other two constraints being satisfied.

A trained model k consisted of a single vector β_k with length 135: slope coefficients for each of the 134 sensors together with an intercept coefficient. We used these trained models to make predictions as to whether unlabeled MEG data corresponded to a neural representation of visual object *k*. Each time point was treated independently. At each time point in the unlabeled data, the data vector over sensors was multiplied by β_k and transformed by a sigmoid to obtain a predicted probability for visual object *k*. This procedure yielded six probabilities at each time point; so for each trial we obtained a matrix X with six columns and as many rows as time bins in the trial.

Sequenceness Measure

Inspired by cross-correlation measures used in the analysis of spike data from rodent hippocampus (e.g., Hoffman and McNaughton, 2002), we used the matrix X to calculate a "sequenceness" measure. Sequenceness operationalized the degree to which decoded MEG activity tended to follow the transition matrix of the task systematically in either a forward or reverse direction. For example, in the task, state S_1 admitted a transition to state S_2 (but not S_3, S_4, or S_6; cf. Figure 1A). If X contained forward sequences, then the decoded probability of S_1 at time T should be correlated with the decoded probability of S_2 at time $T + t$, where *t* defines a lag between neural state representations. Meanwhile, reverse sequences would be expected to support the opposite decoding order.

The sequenceness measure was calculated independently for each possible lag t. We first multiplied X by the transition matrix of the task (assuming equal probabilities for the two possible actions from each state) to obtain X^F and then calculated the cross-correlation at varying time-lags between column *i* of X and column *i* of X^F. This produced six correlations for each possible time-lag. These six correlations were averaged to obtain a single number representing the overall forward sequenceness at that time-lag. In parallel, we also multiplied X by the reverse transition matrix of the task to obtain X^R and again calculated the cross-correlation at varying time-lags between X and X^R. This yielded the reverse sequenceness at each time-lag. We finally subtracted the reverse sequenceness from the forward sequenceness at each time-lag.

As a parametric test for the difference of sequence effect from zero, we estimated a mixed model with only intercept (and no slope) terms.

$$y_i = \beta + b_k + error_i,$$

where y_i was the sequenceness on trial *i*, β was the fixed effect intercept, *k* was the participant on trial *i*, and b_k was the random effect intercept for participant *k*. b_k were assumed to be normally distributed with variance σ. The model was estimated using Matlab's fitlme function.

We also protected the statistical inference using non-parametric permutation tests involving all possible ways of scrambling the six state labels. There were 30 unique permutations of the state labels up to symmetries in the transition matrix of the task. One of these was the identity permutation and one was the reverse-order permutation. For non-parametric significance testing, we therefore used the peak of the absolute value of the other 28 unique permutations as a threshold, at an approximate empirical two-tailed p value of $1/28 \approx 0.036$.

Although sample order is sometimes shuffled in rodent electrophysiology experiments, we found in simulations that the substantial autocorrelation in the MEG time series meant that shuffling sample order led to very high false positive rates (Figure S4). Shuffling state identity is a more conservative measure, which allowed us to reject the null hypothesis that the MEG time series had no relationship to the transition structure of the task.

Importantly, the statistical tests protect against the possibility of systematic dynamics in brain activity (for example, anterior to posterior sweeps of activity) leading to false detection of sequences. Any such activity not reflecting the transition structure of the task would, by definition, be unbiased in whether it appeared to progress forward or backward through the task transitions.

Up to 60 s of MEG data per trial were analyzed for sequenceness, potentially raising a question of whether false positives could arise through the sheer amount of data analyzed. However, this can be ruled out. The cross-correlation analysis does not report the total number of sequences, but the density of sequences per time, meaning that analyzing a longer period of MEG data would not inflate the sequenceness measure. Also, the permutation test used scales to the amount of data, since each permutation is calculated on the full data.

Testing for Length-*n* Sequences

We also computed the extent to which neural sequences followed multiple consecutive steps with the same state-to-state lag, using a similar procedure as described above. Multiplying X by the task's transition matrix twice, we obtained X^{FF}. We then element-wise multiplied column *i* of X, column *i* of X^F shifted by lag *t*, and column *i* of X^{FF} shifted by lag 2*t* and summed the product. For each lag *t*, this yielded six numbers akin to cross-correlations except for being un-normalized. These six numbers were averaged to obtain a single number representing the overall propensity for two forward steps to occur consecutively (which we call "length 3" sequences because they consist of three states). An analogous procedure applied to reverse transitions. This process could be generalized to any number of steps. This method explicitly asks whether all states in a length-*n* sequence occur and is not sensitive to sequences that skip intermediate states.

All summary statistics are given as mean ± SE, unless otherwise stated. Median and IQR are given when the underlying distribution is substantially non-Gaussian.

SUPPLEMENTAL INFORMATION

Supplemental Information includes Supplemental Experimental Procedures and five figures and can be found with this article online at http://dx.doi.org/10.1016/j.neuron.2016.05.028.

AUTHOR CONTRIBUTIONS

Z.K.-N., P.D., and R.J.D. designed the study and prepared the manuscript. Z.K.-N. and M.E. acquired the data. Z.K.-N. and P.D. analyzed the data.

ACKNOWLEDGMENTS

This work was supported by the Wellcome Trust (R.J.D. Senior Investigator Award 098362/Z/12/Z). Z.K.-N. was funded by the Max Planck Society. The Wellcome Trust Centre for Neuroimaging was supported by core funding from the Wellcome Trust 091593/Z/10/Z. P.D. was supported by the Gatsby Charitable Foundation. We thank Demis Hassabis, Quentin Huys, Gareth

Barnes, Marc Guitart-Masip, Will Penny, Anna Jafarpour, David Redish, and Tim Behrens for helpful discussions and Matthjis van der Meer for comments on an earlier version of this manuscript.

Received: December 14, 2015
Revised: March 15, 2016
Accepted: May 17, 2016
Published: June 16, 2016

REFERENCES

Allen, T.A., Salz, D.M., McKenzie, S., and Fortin, N.J. (2016). Nonspatial Sequence Coding in CA1 Neurons. J. Neurosci. *36*, 1547–1563.

Buckner, R.L., and Carroll, D.C. (2007). Self-projection and the brain. Trends Cogn. Sci. *11*, 49–57.

Buzsáki, G., and Moser, E.I. (2013). Memory, navigation and theta rhythm in the hippocampal-entorhinal system. Nat. Neurosci. *16*, 130–138.

Carr, M.F., Jadhav, S.P., and Frank, L.M. (2011). Hippocampal replay in the awake state: a potential substrate for memory consolidation and retrieval. Nat. Neurosci. *14*, 147–153.

Cheng, S., and Frank, L.M. (2008). New experiences enhance coordinated neural activity in the hippocampus. Neuron *57*, 303–313.

Collin, S.H.P., Milivojevic, B., and Doeller, C.F. (2015). Memory hierarchies map onto the hippocampal long axis in humans. Nat. Neurosci. *18*, 1562–1564.

Cornwell, B.R., Johnson, L.L., Holroyd, T., Carver, F.W., and Grillon, C. (2008). Human hippocampal and parahippocampal theta during goal-directed spatial navigation predicts performance on a virtual Morris water maze. J. Neurosci. *28*, 5983–5990.

Dalal, S., Jerbi, K., Bertrand, O., Adam, C., Ducorps, A., Schwartz, D., Martinerie, J., and Lachaux, J.-P. (2013). Simultaneous MEG-intracranial EEG: new insights into the ability of MEG to capture oscillatory modulations in the neocortex and the hippocampus. Epilepsy Behav. *28*, 288–292.

Danker, J.F., and Anderson, J.R. (2010). The ghosts of brain states past: remembering reactivates the brain regions engaged during encoding. Psychol. Bull. *136*, 87–102.

Davidson, T.J., Kloosterman, F., and Wilson, M.A. (2009). Hippocampal replay of extended experience. Neuron *63*, 497–507.

Diba, K., and Buzsáki, G. (2007). Forward and reverse hippocampal place-cell sequences during ripples. Nat. Neurosci. *10*, 1241–1242.

Dragoi, G., and Tonegawa, S. (2011). Preplay of future place cell sequences by hippocampal cellular assemblies. Nature *469*, 397–401.

Eichenbaum, H., and Cohen, N.J. (2014). Can we reconcile the declarative memory and spatial navigation views on hippocampal function? Neuron *83*, 764–770.

Eschenko, O., Ramadan, W., Mölle, M., Born, J., and Sara, S.J. (2008). Sustained increase in hippocampal sharp-wave ripple activity during slow-wave sleep after learning. Learn. Mem. *15*, 222–228.

Euston, D.R., Tatsuno, M., and McNaughton, B.L. (2007). Fast-forward playback of recent memory sequences in prefrontal cortex during sleep. Science *318*, 1147–1150.

Foster, D.J., and Wilson, M.A. (2006). Reverse replay of behavioural sequences in hippocampal place cells during the awake state. Nature *440*, 680–683.

Foster, D.J., and Wilson, M.A. (2007). Hippocampal theta sequences. Hippocampus *17*, 1093–1099.

Girardeau, G., Benchenane, K., Wiener, S.I., Buzsáki, G., and Zugaro, M.B. (2009). Selective suppression of hippocampal ripples impairs spatial memory. Nat. Neurosci. *12*, 1222–1223.

Guitart-Masip, M., Barnes, G.R., Horner, A., Bauer, M., Dolan, R.J., and Duzel, E. (2013). Synchronization of medial temporal lobe and prefrontal rhythms in human decision making. J. Neurosci. *33*, 442–451.

Gupta, A.S., van der Meer, M.A.A., Touretzky, D.S., and Redish, A.D. (2010). Hippocampal replay is not a simple function of experience. Neuron *65*, 695–705.

Gupta, A.S., van der Meer, M.A.A., Touretzky, D.S., and Redish, A.D. (2012). Segmentation of spatial experience by hippocampal θ sequences. Nat. Neurosci. *15*, 1032–1039.

Hämäläinen, M., Hari, R., Ilmoniemi, R.J., Knuutila, J., and Lounasmaa, O.V. (1993). Magnetoencephalography–theory, instrumentation, and applications to noninvasive studies of the working human brain. Rev. Mod. Phys. *65*, 413–497.

Hoffman, K.L., and McNaughton, B.L. (2002). Coordinated reactivation of distributed memory traces in primate neocortex. Science *297*, 2070–2073.

Huys, Q.J., Eshel, N., O'Nions, E., Sheridan, L., Dayan, P., and Roiser, J.P. (2012). Bonsai trees in your head: how the pavlovian system sculpts goal-directed choices by pruning decision trees. PLoS Comput. Biol. *8*, e1002410.

Huys, Q.J., Lally, N., Faulkner, P., Eshel, N., Seifritz, E., Gershman, S.J., Dayan, P., and Roiser, J.P. (2015). Interplay of approximate planning strategies. Proc. Natl. Acad. Sci. USA *112*, 3098–3103.

Jadhav, S.P., Kemere, C., German, P.W., and Frank, L.M. (2012). Awake hippocampal sharp-wave ripples support spatial memory. Science *336*, 1454–1458.

Jensen, O., and Lisman, J.E. (2005). Hippocampal sequence-encoding driven by a cortical multi-item working memory buffer. Trends Neurosci. *28*, 67–72.

Ji, D., and Wilson, M.A. (2007). Coordinated memory replay in the visual cortex and hippocampus during sleep. Nat. Neurosci. *10*, 100–107.

Johnson, A., and Redish, A.D. (2007). Neural ensembles in CA3 transiently encode paths forward of the animal at a decision point. J. Neurosci. *27*, 12176–12189.

Káli, S., and Dayan, P. (2004). Off-line replay maintains declarative memories in a model of hippocampal-neocortical interactions. Nat. Neurosci. *7*, 286–294.

Karlsson, M.P., and Frank, L.M. (2009). Awake replay of remote experiences in the hippocampus. Nat. Neurosci. *12*, 913–918.

Killian, N.J., Jutras, M.J., and Buffalo, E.A. (2012). A map of visual space in the primate entorhinal cortex. Nature *491*, 761–764.

Kuhl, B.A., and Chun, M.M. (2014). Successful remembering elicits event-specific activity patterns in lateral parietal cortex. J. Neurosci. *34*, 8051–8060.

Kurth-Nelson, Z., Barnes, G., Sejdinovic, D., Dolan, R., and Dayan, P. (2015). Temporal structure in associative retrieval. eLife *4*, e04919.

Lansink, C.S., Goltstein, P.M., Lankelma, J.V., McNaughton, B.L., and Pennartz, C.M.A. (2009). Hippocampus leads ventral striatum in replay of place-reward information. PLoS Biol. *7*, e1000173.

LaValle, S.M. (2006). Planning Algorithms (Cambridge, New York: Cambridge University Press).

Lee, A.K., and Wilson, M.A. (2002). Memory of sequential experience in the hippocampus during slow wave sleep. Neuron *36*, 1183–1194.

Louie, K., and Wilson, M.A. (2001). Temporally structured replay of awake hippocampal ensemble activity during rapid eye movement sleep. Neuron *29*, 145–156.

Milivojevic, B., and Doeller, C.F. (2013). Mnemonic networks in the hippocampal formation: from spatial maps to temporal and conceptual codes. J. Exp. Psychol. Gen. *142*, 1231–1241.

Mnih, V., Kavukcuoglu, K., Silver, D., Rusu, A.A., Veness, J., Bellemare, M.G., Graves, A., Riedmiller, M., Fidjeland, A.K., Ostrovski, G., et al. (2015). Human-level control through deep reinforcement learning. Nature *518*, 529–533.

Muller, R.U., Stead, M., and Pach, J. (1996). The hippocampus as a cognitive graph. J. Gen. Physiol. *107*, 663–694.

Nádasdy, Z., Hirase, H., Czurkó, A., Csicsvari, J., and Buzsáki, G. (1999). Replay and time compression of recurring spike sequences in the hippocampus. J. Neurosci. *19*, 9497–9507.

Nyberg, L., Habib, R., McIntosh, A.R., and Tulving, E. (2000). Reactivation of encoding-related brain activity during memory retrieval. Proc. Natl. Acad. Sci. USA *97*, 11120–11124.

O'Keefe, J., and Nadel, L. (1978). The hippocampus as a cognitive map (Oxford University Press).

Ólafsdóttir, H.F., Barry, C., Saleem, A.B., Hassabis, D., and Spiers, H.J. (2015). Hippocampal place cells construct reward related sequences through unexplored space. eLife *4*, e06063.

Pezzulo, G., van der Meer, M.A.A., Lansink, C.S., and Pennartz, C.M.A. (2014). Internally generated sequences in learning and executing goal-directed behavior. Trends Cogn. Sci. *18*, 647–657.

Pfeiffer, B.E., and Foster, D.J. (2013). Hippocampal place-cell sequences depict future paths to remembered goals. Nature *497*, 74–79.

Polyn, S.M., Natu, V.S., Cohen, J.D., and Norman, K.A. (2005). Category-specific cortical activity precedes retrieval during memory search. Science *310*, 1963–1966.

Redish, A.D. (1999). Beyond the Cognitive Map (MIT Press).

Riggs, L., Moses, S.N., Bardouille, T., Herdman, A.T., Ross, B., and Ryan, J.D. (2009). A complementary analytic approach to examining medial temporal lobe sources using magnetoencephalography. Neuroimage *45*, 627–642.

Schapiro, A.C., Kustner, L.V., and Turk-Browne, N.B. (2012). Shaping of object representations in the human medial temporal lobe based on temporal regularities. Curr. Biol. *22*, 1622–1627.

Schmidt, B., and Redish, A.D. (2013). Neuroscience: Navigation with a cognitive map. Nature *497*, 42–43.

Shapiro, M.L., and Eichenbaum, H. (1999). Hippocampus as a memory map: synaptic plasticity and memory encoding by hippocampal neurons. Hippocampus *9*, 365–384.

Shohamy, D., and Daw, N.D. (2015). Integrating memories to guide decisions. Curr. Opin. Behav. Sci. *5*, 85–90.

Siapas, A.G., and Wilson, M.A. (1998). Coordinated interactions between hippocampal ripples and cortical spindles during slow-wave sleep. Neuron *21*, 1123–1128.

Sirota, A., Csicsvari, J., Buhl, D., and Buzsáki, G. (2003). Communication between neocortex and hippocampus during sleep in rodents. Proc. Natl. Acad. Sci. USA *100*, 2065–2069.

Skaggs, W.E., and McNaughton, B.L. (1996). Replay of neuronal firing sequences in rat hippocampus during sleep following spatial experience. Science *271*, 1870–1873.

Skaggs, W.E., McNaughton, B.L., Wilson, M.A., and Barnes, C.A. (1996). Theta phase precession in hippocampal neuronal populations and the compression of temporal sequences. Hippocampus *6*, 149–172.

Stephen, J.M., Ranken, D.M., Aine, C.J., Weisend, M.P., and Shih, J.J. (2005). Differentiability of simulated MEG hippocampal, medial temporal and neocortical temporal epileptic spike activity. J. Clin. Neurophysiol. *22*, 388–401.

Sutton, R.S. (1991). Dyna, an Integrated Architecture for Learning (Planning, and Reacting).

Takahashi, S. (2013). Hierarchical organization of context in the hippocampal episodic code. eLife *2*, e00321.

Takahashi, S. (2015). Episodic-like memory trace in awake replay of hippocampal place cell activity sequences. eLife *4*, e08105.

Tanaka, K.Z., Pevzner, A., Hamidi, A.B., Nakazawa, Y., Graham, J., and Wiltgen, B.J. (2014). Cortical representations are reinstated by the hippocampus during memory retrieval. Neuron *84*, 347–354.

Tavares, R.M., Mendelsohn, A., Grossman, Y., Williams, C.H., Shapiro, M., Trope, Y., and Schiller, D. (2015). A Map for Social Navigation in the Human Brain. Neuron *87*, 231–243.

Tolman, E.C. (1948). Cognitive maps in rats and men. Psychol. Rev. *55*, 189–208.

van der Meer, M.A.A., and Redish, A.D. (2009). Covert Expectation-of-Reward in Rat Ventral Striatum at Decision Points. Front. Integr. Nuerosci. *3*, 1.

van der Meer, M.A.A., Johnson, A., Schmitzer-Torbert, N.C., and Redish, A.D. (2010). Triple dissociation of information processing in dorsal striatum, ventral striatum, and hippocampus on a learned spatial decision task. Neuron *67*, 25–32.

van der Meer, M., Kurth-Nelson, Z., and Redish, A.D. (2012). Information processing in decision-making systems. Neuroscientist *18*, 342–359.

Wierzynski, C.M., Lubenov, E.V., Gu, M., and Siapas, A.G. (2009). State-dependent spike-timing relationships between hippocampal and prefrontal circuits during sleep. Neuron *61*, 587–596.

Wikenheiser, A.M., and Redish, A.D. (2013). The balance of forward and backward hippocampal sequences shifts across behavioral states. Hippocampus *23*, 22–29.

Wikenheiser, A.M., and Redish, A.D. (2015). Hippocampal theta sequences reflect current goals. Nat. Neurosci. *18*, 289–294.

Wimmer, G.E., and Shohamy, D. (2012). Preference by association: how memory mechanisms in the hippocampus bias decisions. Science *338*, 270–273.

Neuron, Volume *91*

Supplemental Information

Fast Sequences of Non-spatial
State Representations in Humans

Zeb Kurth-Nelson, Marcos Economides, Raymond J. Dolan, and Peter Dayan

Supplemental Figures

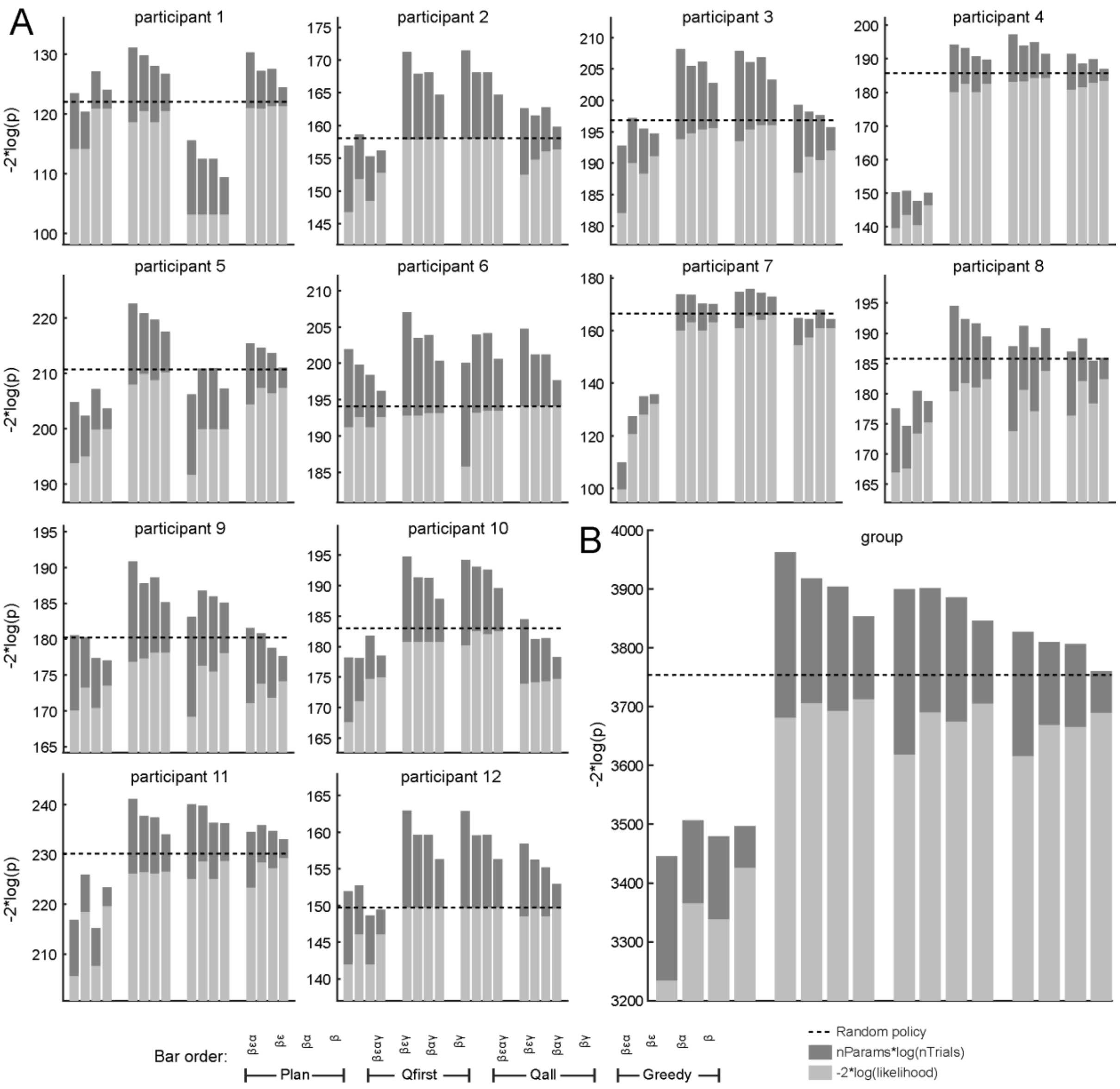

[Related to Figure 1]

Figure S1 - Behavioral model comparison. Four different models were fitted to behavioral choice data: "Plan", "Qfirst", "Qall" and "Greedy". Each model is described in the Experimental Procedures. Each model always had an inverse temperature parameter β, and the Q-learning models always had a discount rate parameter γ. There were two optional parameters in each model: a lapse rate ε and a learning rate α. Each combination of including or excluding ε and α was explored for each model. Light gray bars show two times the negative log of maximum likelihood. Dark gray bars show the BIC model complexity penalty, which depends on the number of parameters. The sum of these two quantities is an approximation of two times the total negative log evidence for each model. **A,** In 11/12 participants, the planning model outperformed the simple RL models. The order of the bars in each plot is the same as in Figure S2. Note that the evidence for the random policy depended on the number of trials completed, and so differed between participants. **B,** At the group level, the planning model was favored over the best simple RL model by 157 log units of evidence. The simple RL models did not perform better than chance, after taking into account the complexity penalty.

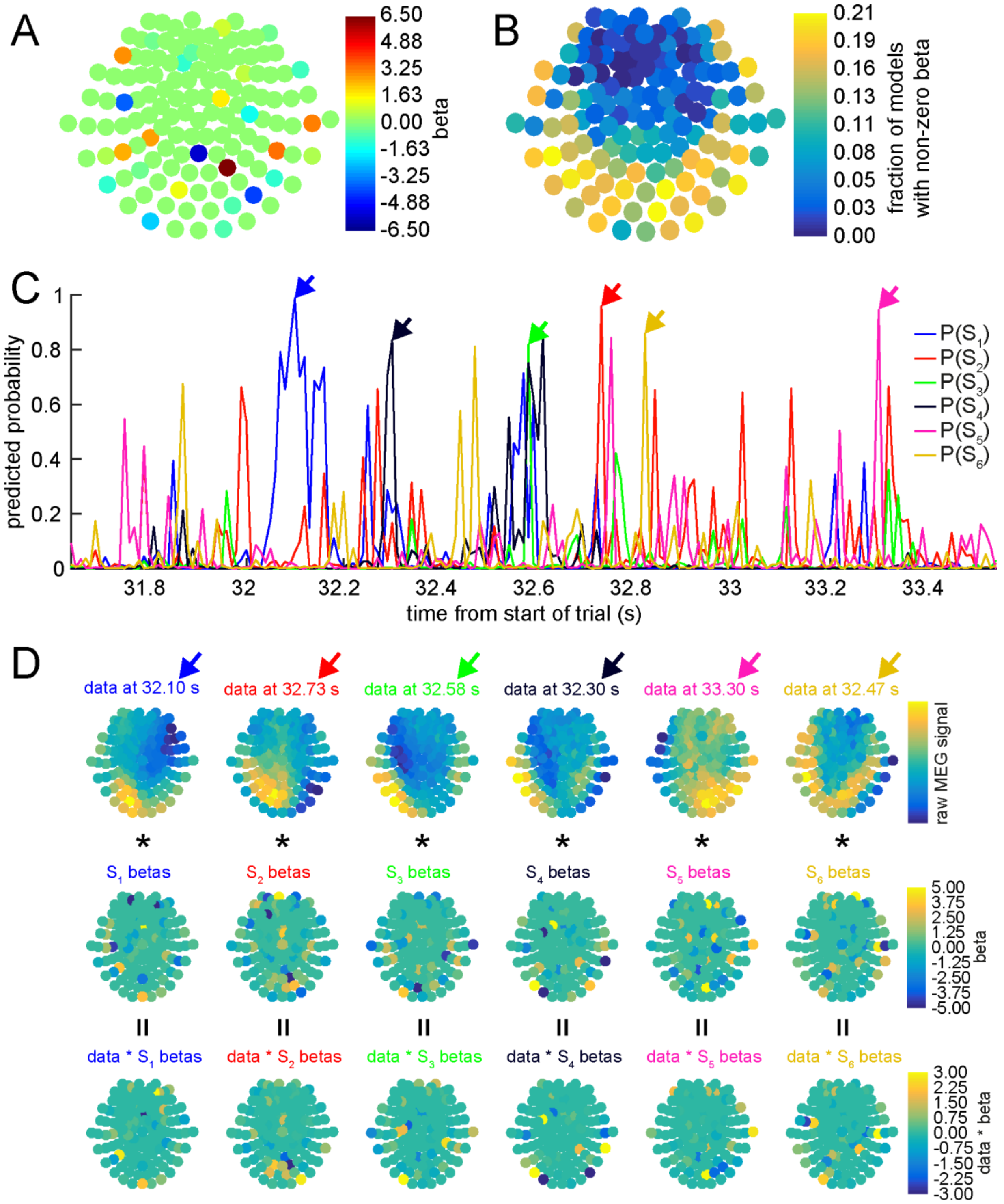

[Related to Figure 2]

Figure S2 – Decoding methods. For each session, six lasso-regularized logistic regression models were trained, one for each visual object in the primary task. **A,** The lasso penalty encouraged sparsity, so the majority of coefficients in each trained model were zero. One example trained model is shown here (intercept not shown). **B,** These models tended to select mostly occipital and temporal sensors. Of the 108 models used in analysis (18 sessions times 6 models), this plot shows the fraction where each sensor was non-zero. **C,** An example of the six probability time series output by the six regression models, over a short segment of time. Six time points, indicated by colored arrows, were selected for display in D. **D,** The top row shows the raw MEG data at the six time points indicated by arrows in C. The middle row shows the learned betas for the classifier whose output is high at this

time. The bottom row shows the element-wise product of the data at that time point with the illustrated classifier. The mean across sensors of these products, plus the intercept of the model, was the value *x* used to generate a probability through the transformation 1 / (1 + exp(-*x*)).

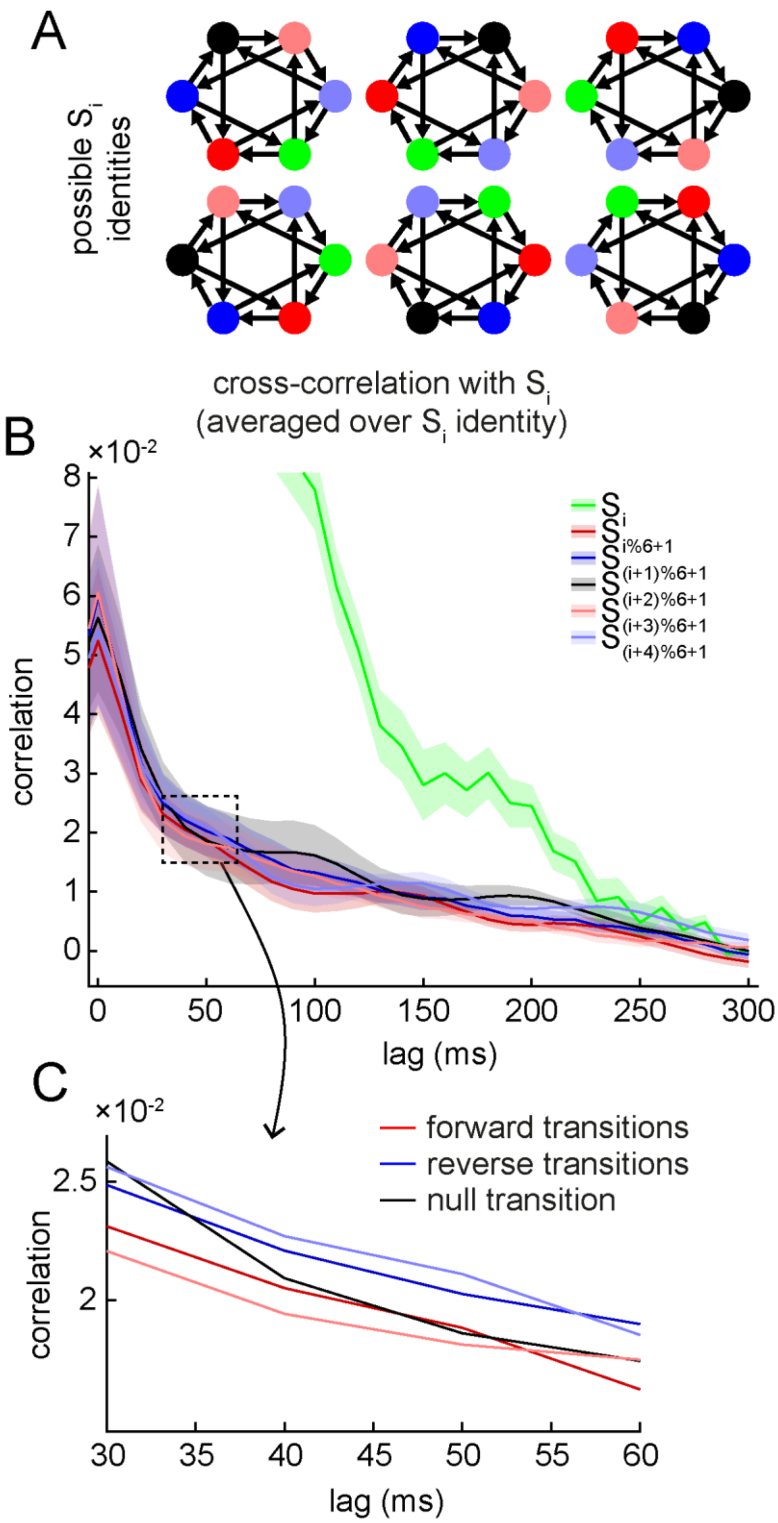

[Related to Figure 3]

Figure S3 – Autocorrelation in classifier outputs. Cross-correlations between pairs of states had a strong peak near zero lag due to autocorrelation in the underlying MEG signal. **A,** Symmetries over

state relationships, defining the color mapping used in B and C. **B,** Cross-correlations were calculated for each ordered pair of states, yielding 36 cross-correlations. These were collapsed by averaging over equivalent transitions (under the symmetries shown in A; for example, S_1->S_2 was equivalent to S_2->S_3), giving six cross-correlations, which are shown here. At 40 ms lag, the reverse transitions (blue and light blue) had stronger correlation than the forward transitions (red and light red). The reverse transitions also had stronger correlation than the null transitions (black), but this was not statistically significant. The identity transition (green) had strong autocorrelation. **C,** Magnification of lags from 30 to 60 ms. The difference between the blue traces and the red traces reflects the sequenceness effect shown in Figure 3 in the main text.

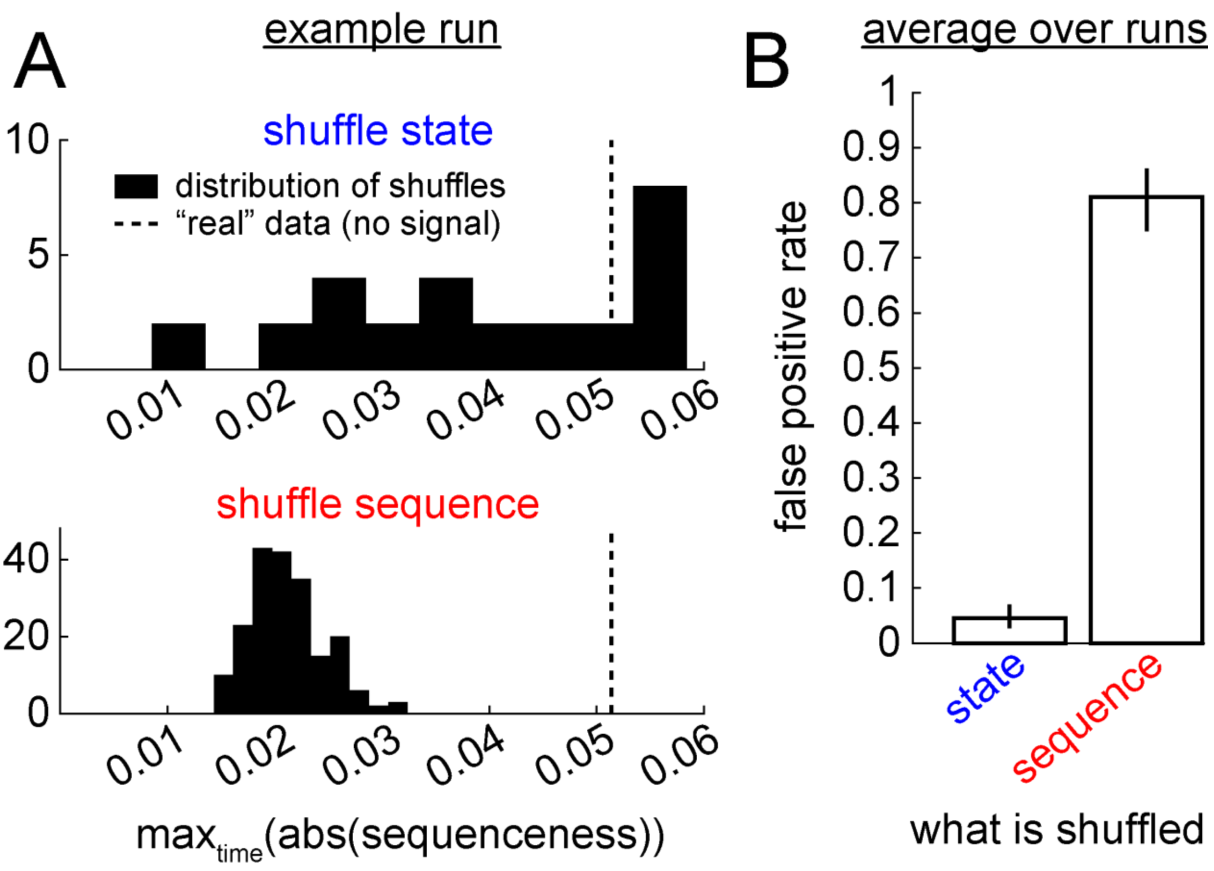

[Related to Figure 3]
Figure S4 - Shuffling sequence produces false positives. We simulated six autocorrelated time series of classifier predictions in which there was no true sequenceness. **A,** We applied our sequenceness analysis method to this synthetic data to obtain sequenceness for lags between 10 and 600 ms, in 10 ms increments. The maximum of the absolute value of these sequenceness measures over all lags is shown as a dashed line. We then applied the same state shuffling procedure used on our MEG data to obtain a null distribution of maximum statistics (black histogram, top panel). The "real" data did not exceed either the maximum of this distribution (the criterion we used on our MEG data), or the 95th percentile, consistent with the fact that there was no sequenceness in the simulated data. However, when we used a shuffling procedure that shuffled the temporal sequence of the data instead of shuffling state identities, the "real" data exceeded the maximum of the null distribution, because shuffling sequence creates an artificially narrow null distribution. **B,** We repeated the procedure in A 400 times for each shuffling method. The "real" data exceeded the null distribution of the state shuffling method in 4.5% (95% binomial CI [2.7%, 7.0%]) of the repetitions. However, despite there being no signal in the data, the "real" data exceeded the null distribution of the sequence shuffling method in 81% (95% binomial CI [75%, 86%]) of the repetitions.

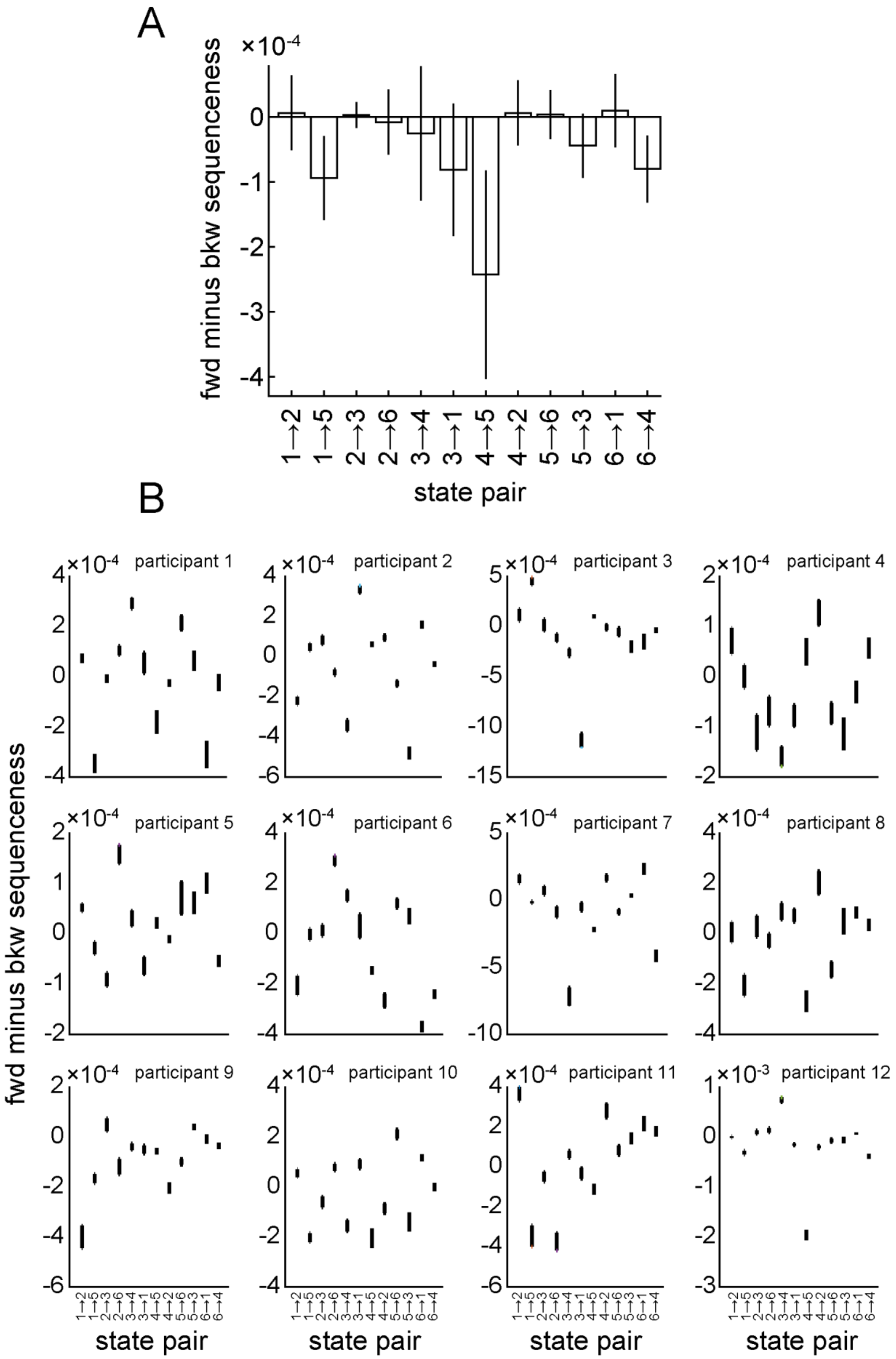

[Related to Figure 3]

Figure S5 - Sequenceness at 40 ms for each state pair. For each ordered pair of states having a transition between them in the task (shown on the x-axis of each panel), we subtracted the cross-correlation between these states at -40 ms from that at +40 ms. This subtracted cross-correlation is shown on the y-axis in all panels. **A,** In the group average, there was no reliable difference between state pairs by one-way ANOVA ($F(11,132)=0.92$, $p=0.52$). This was expected, given that the visual objects associated with the states were randomized across participants. **B,** In each individual participant, there was a significant difference between state pairs that was reliable across trials (ANOVA F-statistics ranged from 8 to 84).

Supplemental Experimental Procedures

Task

From a participant's point of view, each trial involved three phases: planning, move pre-entry, and move execution. During planning, participants saw in text the names of the starting state and the two neg states for that trial. Participants had up to 60 seconds to plan their moves, but could also press a button to enter their moves sooner. After the 60 seconds or this button press, there was a 1-second warning to signal the start of the move pre-entry phase. During move pre-entry, participants were required to enter their chosen sequence of four choices rapidly, while receiving no visual feedback except the corresponding arrows (up and down) appearing to reflect their move selections. If they took longer than 1 second per move, the trial was aborted with a fixed -10 pence loss. One percent of all trials was aborted due to time-outs.

Next, during move execution participants were required to play out the sequence of four moves they selected during pre-entry. On each move, the button corresponding to the unchosen option (up or down) was deactivated, so there was no possibility of deviating from the pre-entered sequence. During execution, the visual objects corresponding to each state were shown as the participant moved from state to state. The transitions between objects were visually cross-faded with a blend ratio that changed linearly from 100%/0% to 0%/100% over 350 ms. The current reward values of each object were also shown, and the cumulative reward total for the trial was updated as each new state was reached. If a state was neg, the cumulative reward total would first reflect the addition of the state's reward value, and then the text "neg" would appear and the displayed cumulative reward total would flip its sign.

The transition structure of the maze was identical for all subjects. The assignment of "up" and "down" to each transition was randomized between subjects. The pseudo-random sequence of rewards, starting states, and negative states was also identical for all sessions (although the number of trials completed varied between sessions due to the self-paced nature of the task) and was optimized using a genetic algorithm. The primary goal of optimization was to arrange it that on nearly half of trials, optimal and greedy choices differed for the first choice in the sequence, where greedy choice means selecting the highest-value next state. (If this target had been more than a half, subjects could have employed the shortcut of doing the opposite of the greedy strategy.) A secondary goal of optimization was avoiding degenerate cases such as repeating the same starting conditions.

At the end of all training and scanning sessions, participants received their earnings on the task, which ranged from £-0.60 to £1.76, plus £10 for each day of behavioral training and £15 for each day of scanning. The amount of money that could be earned varied substantially from trial to trial, so in the paper per-trial earnings are reported as $E - E_{rand}$, where E was the actual earnings on the trial, E_{rand} was the expected earnings under random play on this trial (both in units of pence).

Behavioral training

Participants received either two or three days of training on the task structure before scanning. Training progressed until a participant reached 100% performance on the final set of quizzes used for training. Training started by introducing the six visual objects and quizzing participants about single transitions (e.g., if you are in "tree" and you choose "down", which state will you arrive at?). Quizzes were then extended to two-move sequences and four-move sequences. Finally, rewards were introduced, and participants were quizzed about how much money they would earn by taking specified sequences of four moves.

The visual objects and transition structure used during training were preserved for each subject into the actual experiment, but the rewards used during training were independent of the rewards used in

the actual experiment. All training was automated in MATLAB/Cogent to minimize experimenter interaction.

Behavioral models

We fit four models of choice behavior. "Plan" used an optimal full-depth tree search to calculate the value of each of the 16 possible sequences of four moves on a trial. This planning depended on tracking the drifting rewards associated with each state. The model allowed for gradual learning about the drifting rewards, following a learning rate (α). Each time the current reward R_i for state *i* was revealed, the tracked reward ρ_i was updated:

$$\rho_i \leftarrow \rho_i + \alpha \cdot (R_i - \rho_i)$$

The values, V, of each of the 16 possible sequences of four moves were calculated optimally and transformed to choice probabilities with a sigmoidal link function parameterized by inverse temperature (β), and lapse rate (ε):

$$P_i = \varepsilon + (1 - 16\varepsilon) \cdot \frac{e^{\beta \cdot V_i}}{\sum_i e^{\beta \cdot V_i}}$$

To evaluate whether participants were expressing knowledge of the task's transition structure in their choice behavior, we compared this optimal model against simpler models. The simplest was a Q-learner, "Qfirst", with no knowledge of the transition structure. Because there were only on the order of 35 trials per session, it would be impossible to learn an action value representation over the entire space of 6 starting states × 16 actions. Meanwhile, it would be impossible to use the same set of Q-values to make predictions from future states without knowing the transition structure. "Qfirst" tackled these problems by only making predictions about the first move in each trial, reducing the number of Q-values to 6 × 2 = 12. Based on the final reward *M* obtained at the end of each trial, the Q-value for the chosen first action *c* was updated:

$$Q_{s,c} \leftarrow Q_{s,c} + \alpha \cdot \left(\gamma \cdot \left(M + \max_a Q_{s',a}\right) - Q_{s,c}\right)$$

where α was a learning rate, γ was a discount rate, *s* was the starting state for the trial, and the action *c* led to state *s*'. The first move on each trial was selected by softmax over the Q-values of the two possible actions:

$$P_i = \varepsilon + (1 - 2\varepsilon) \cdot \frac{e^{\beta \cdot Q_{s,i}}}{\sum_i e^{\beta \cdot Q_{s,i}}}$$

The remaining three moves in the trial were selected uniformly at random.

The third model, "Qall", relaxed the requirement that the agent have no knowledge of the transition structure. By relaxing this requirement we allowed the model to make predictions about all four moves in each trial. The best action (i.e., up or down) for move 2, 3 or 4 in a trial depended on what state the agent occupied based on its earlier moves in the trial. The model selected moves 2, 3 and 4 based on the participant's actually chosen, rather than the model's preferred, earlier moves, which allowed the model to make predictions about subsequent moves even if it mispredicted the early moves. Q-value updating and action selection worked the same as in Qfirst, except that Q-values for all four chosen moves were updated, and all four moves were selected (the total probability for a trial was the product of the probabilities for each of these moves).

The fourth model, "Greedy", implemented another strategy we considered *a priori* plausible. First, it tracked the reward values ρ_i for each of the six states, as in the Plan model. Second, it chose greedily at each move the state that led to the largest cumulative total *after that move*, taking negs into account:

$$V_{up} = -1^{neg_{up}} \cdot (CR + \rho_{up})$$

where neg_{up} was a binary variable indicating whether the state that would be reached by taking the up action was neg on this trial, CR was the cumulative reward gained on the trial before this move, and ρ_{up} was the reward value of the state that would be reached by taking the up action. Greedy therefore also used knowledge of the task's transition structure. Action selection was again by softmax, between V_{up} and V_{down}.

The parameters of all the models were fit using a genetic algorithm ('ga' in Matlab) to maximize the likelihood of the data. To guard against local minima, ga was run three times and the best run selected (usually all three runs were very similar).

Model comparison was performed using the Bayesian Information Criterion (BIC). For each participants and for each model, the log likelihood L was evaluated at the best fitting parameters. The BIC score for that participant for that model was

$$-2 \log L + n_p \log n_t$$

where n_p was the number of free parameters for that model, and n_t was the number of trials for that participant. BIC scores were summed over participants for group-level model comparison.

MEG acquisition and pre-processing

MEG was recorded continuously at 600 samples/second using a whole-head 275-channel axial gradiometer system (CTF Omega, VSM MedTech, Canada), while participants sat upright inside the scanner. Continuous head localization was recorded with three fiducial coils at the nasion, left pre-auricular, and right pre-auricular points. The task script sent synchronizing triggers (outportb in Cogent) which were written to the MEG data file. Timings were corrected for approximately one frame (1/60 s) of lag between triggers and refreshing of the projected image, measured using a photodiode outside the task. A projector displayed the task on a screen ~80 cm in front of the participant. Participants made responses on three buttons (called "up", "down" and "advance") of a button box using the fingers they found most comfortable.

Raw data in CTF .meg4 format were read into MATLAB arrays using the function spm_eeg_convert from SPM12 (Wellcome Trust Centre for Neuroimaging, University College London). All subsequent analysis was performed using built-in functions in MATLAB R2015a/b, without SPM. The data were first resampled from 600 Hz to 100 Hz to conserve processing time and improve signal to noise ratio. Thus, data samples used for analysis were spaced every 10 ms. All data were then high-pass filtered at 0.5 Hz using a first-order IIR filter to remove slow drift.

Two approaches were used to reject artifacts. First, we excluded all sensors that had more than 10000 samples of detected eyeblink across all sessions. This also minimized eye movement artifacts. 134 sensors were left included in the analysis. Second, we rejected any data that exceeded seven units of mean absolute difference, plus ten samples before and after. Finally, as mentioned above, two sessions were entirely excluded due to large artifacts that could not be easily rejected according to these methods. All rejection criteria were fixed before the start of sequence analysis.

All analyses were performed directly on the filtered, cleaned MEG signal, consisting of a length 134 vector of samples every 10 ms, in units of femtoTesla. No time-frequency decomposition, baseline correction, source reconstruction, or other pre-processing was used.

Multivariate MEG analysis

Logistic regression models were trained using MATLAB's function lassoglm, with the arguments 'binomial', ('Alpha', 1), and ('Lambda', Lp) where Lp was the lasso penalty whose value was determined through a cross-validation procedure. A trained model k consisted of a single vector $\boldsymbol{\beta}_k$ with length 135: slope coefficients for each of the 134 sensors together with an intercept coefficient. For both training and testing regression models, all input data were scaled such that the 95th percentile of the absolute value of each channel was equal to 1.

For model k, the training data labelled as "1" were the trials in which visual object k was presented. The training data labelled as "0" were trials in which any of the other five objects were presented, as well as a set of "null" data. The null data were the same for each of the six regression models, and consisted of the data immediately before the onset of each visual object. Thus, each model was trained on approximately 16 positive examples and 183 negative examples (and 100 of these ~183 were the same for all models). Models were validated on labelled data using leave-one-out cross validation.

To estimate the number of meaningfully independent components in the data, we performed principal components analysis on the data used to train the classifier (from 200 ms after stimulus onset). We found that the number of components needed to explain 95% of the variance across trials ranged from 6 to 17 between subjects.

Sequenceness Measure

Subtracting reverse from forward sequenceness was motivated by the existence of a strong autocorrelation in the underlying MEG signal at short time-lags (as time lag increased, autocorrelation became negative due to highpass filtering). Because classifier outputs were correlated with one another, each pair of classifier outputs also had a corresponding peak in cross-correlation near zero time lag. Therefore, both forward and reverse sequenceness alone were strongly positive at short time lags (Figure S3). Taking the difference between forward and reverse sequenceness minimized the main effect of autocorrelation while maximizing detectability of a systematic forward or reverse sequence effect. However, it was also possible to compare either forward or reverse transitions against probabilities that would be expected if representations jumped across a transition that did not exist in the task ('null transitions'; for example from S_1, jumping to S_4). Although reverse transitions were greater than null transitions (Figure S3), this difference did not reach significance due to high variability in the differences between cross-correlations of unmatched pairs of states.

For the participants who had two sessions, regression models were trained separately for each session to account for the possibility of different head positions, and sequenceness measures from the two sessions were averaged together before computing group-level statistics.
